# Metapopulation Persistence in Random Fragmented Landscapes

**DOI:** 10.1371/journal.pcbi.1004251

**Published:** 2015-05-20

**Authors:** Jacopo Grilli, György Barabás, Stefano Allesina

**Affiliations:** 1 Department of Physics and Astronomy ‘G. Galilei’, Università di Padova, Padova, Italy; 2 Department of Ecology & Evolution, University of Chicago, Chicago, Illinois, United States of America; 3 Computation Institute, University of Chicago, Chicago, Illinois, United States of America; The Pennsylvania State University, UNITED STATES

## Abstract

Habitat destruction and land use change are making the world in which natural populations live increasingly fragmented, often leading to local extinctions. Although local populations might undergo extinction, a metapopulation may still be viable as long as patches of suitable habitat are connected by dispersal, so that empty patches can be recolonized. Thus far, metapopulations models have either taken a mean-field approach, or have modeled empirically-based, realistic landscapes. Here we show that an intermediate level of complexity between these two extremes is to consider random landscapes, in which the patches of suitable habitat are randomly arranged in an area (or volume). Using methods borrowed from the mathematics of Random Geometric Graphs and Euclidean Random Matrices, we derive a simple, analytic criterion for the persistence of the metapopulation in random fragmented landscapes. Our results show how the density of patches, the variability in their value, the shape of the dispersal kernel, and the dimensionality of the landscape all contribute to determining the fate of the metapopulation. Using this framework, we derive sufficient conditions for the population to be spatially localized, such that spatially confined clusters of patches act as a source of dispersal for the whole landscape. Finally, we show that a regular arrangement of the patches is always detrimental for persistence, compared to the random arrangement of the patches. Given the strong parallel between metapopulation models and contact processes, our results are also applicable to models of disease spread on spatial networks.

## Author Summary

Like the hundreds of paintings of water lilies by Monet, any two landscapes in which a metapopulation dwells are different, as the size, shape and location of the patches of suitable habitat (the lilies), distributed over a inhospitable background (the water) vary among landscapes. Yet, as all the paintings depict the same pond in Giverny, different fragmented landscapes could have the same value to a metapopulation. Here we ask what are the key features we should measure to predict persistence of metapopulations inhabiting fragmented landscapes, and show that few quantities determine the fate of metapopulations—so that two very different-looking landscapes could lead to the same likelihood of persistence. We also show that regular arrangements of the patches in space are detrimental for persistence, and that the typical behavior of metapopulations close to extinction is to be mostly localized in a confined region of the landscape.

## Introduction

In an increasingly fragmented and patchy world [1], species survival critically depends on dispersal, as local populations at high risk of extinction could be rescued by immigration from neighboring populations [2]. This intuition forms the core of metapopulation theory: even though local populations occupying patches of suitable habitat might undergo extinction, persistence can be achieved at the metapopulation level—rather than in each patch—as long as individuals can disperse between patches and thus recolonize empty ones [1].

It was Levins [2] who first proposed a species occupancy model (where the goal is to measure the presence/absence of a species in a patch) which assumed infinitely many patches of suitable habitat, all mutually reachable from any other. His work highlighted one of the main features common also to more complex models, namely that persistence is achieved when the colonization rate exceeds the extinction rate (S1 Text).

Hanski & Ovaskainen [3] extended this formulation to realistic landscapes, composed of multiple patches, each having a different “value” (e.g., size, or density of resources), connected by dispersal whose strength depends on the distance between patches. This effectively defines the landscape as a network in which the nodes are the patches, and the weighted edges express the colonization rates [3–7].

When developing a general theory of persistence in fragmented landscapes, we are faced with the problem that no two landscapes are alike. The situation is reminiscent of complex network theory, in which any two food webs, transportation, or gene-regulation networks are different, making it difficult to pinpoint the salient features of each system. In these cases, much progress has been made by contrasting empirical networks with those generated by simple models such as the Erdős-Rényi random graph or the Barabási-Albert model [8]. Our main goal is to propose and study a reference model for metapopulations dispersing in fragmented landscapes.

One could be tempted to simply take the Erdős-Rényi model and apply it to metapopulations [7]. However, this model lacks a fundamental feature of real dispersal networks: two patches close in space are more likely to exchange individuals than two that are far away. A more fruitful avenue is to take *N* patches, distribute them randomly in space, and connect any two patches that are closer than some threshold distance [9]. This defines a so-called Random Geometric Graph [10–12], for which it has been shown that the number of edges per node required to make the graph connected (i.e., the graph is composed of just one “piece”) is much higher than that for Erdős-Rényi graphs, with the two converging for high-dimensional landscapes [13]. However, natural populations exist in low-dimensional environments, with species endemic to the dunes of Lake Michigan [14] experiencing what is effectively a one-dimensional space, and birds nesting in fragmented forests living in a two-dimensional landscape. As such, Erdős-Rényi random graphs are inadequate descriptors of natural dispersal networks [7].

**Fig 1:**
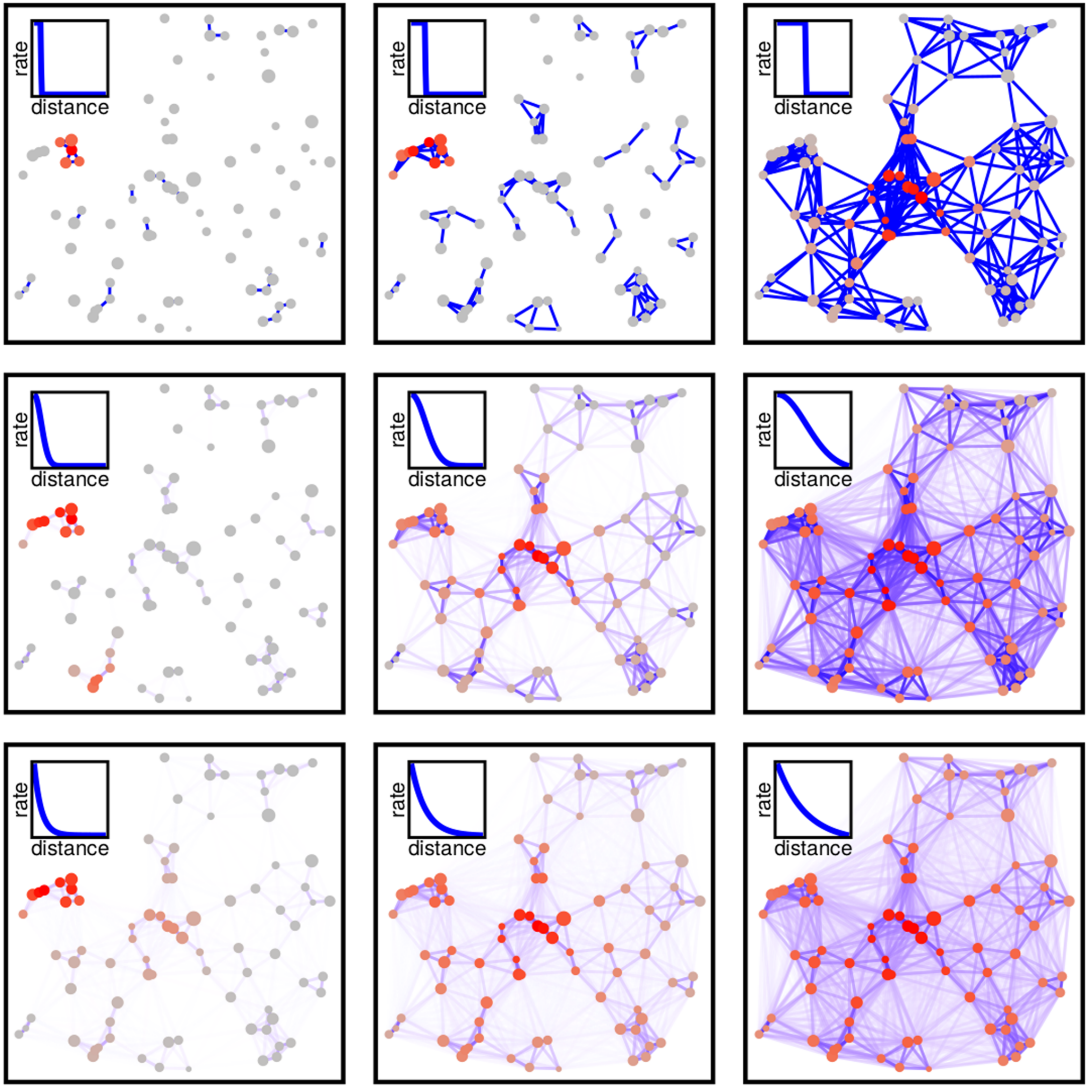
Fragmented landscapes as networks. Different rows represent different dispersal kernels (top to bottom: Rectangular, Gaussian and Exponential, as depicted in the panel insets). Columns represent increasing values of the dispersal length *ξ*. The size of the points stands for the patch value *A*_*i*_, while their color measures the probability of occupancy *p*_*i*_ (gray → low probability, red → high probability). The color of the edges measures their strength (white to blue). In all the panels *δ* = 0.9*λ*, where *λ* is the metapopulation capacity, and *δ* is the background extinction rate (see Eq 3).

Random Geometric Graphs can be generalized even further. Instead of treating the connectedness of two patches in an “either/or” manner, we may think of it as a smooth function of distance. This function is what we refer to as the “dispersal kernel”. Such networks have been introduced in the physics of disordered systems [15] and are called Euclidean Random Matrices (S1 Text). In fact, a Random Geometric Graph is but one special case, in which the dispersal kernel is rectangular (Fig 1, top row), yielding a dispersal rate of either 0 or 1, while generic Euclidean Random Matrices cover the broad spectrum of intermediate cases where dispersal rates vary smoothly with distance (Fig 1, bottom two rows).

Here we use Euclidean Random Matrices to study metapopulations dispersing in random fragmented landscapes. For simplicity, we treat the case of patches that are uniformly distributed in space, and with patch values independently sampled from a random distribution. We derive a condition for metapopulation persistence analytically, highlighting that number of spatial dimensions, number of patches, shape of the dispersal kernel, and the variability in patch value are all key to determining persistence. We also show that a metapopulation can persist in two different regimes, localized and nonlocalized, giving rise to completely different responses to habitat loss. We finally show that arranging the patches in a perfect grid (as often considered in the design of protected areas [16–18]) yields a lower likelihood of persistence compared to the case in which we distribute the patches randomly.

## Methods

### Metapopulation and Susceptible-Infected-Susceptible models

Take *N* patches of suitable habitat, positioned in a landscape. Each patch can be either occupied or unoccupied by the species of interest at time *t*. Empty patches can be colonized through dispersal from occupied patches; occupied patches can become empty following a local extinction event. For simplicity, we assume that both colonization and extinction are independent Poisson processes in continuous time.

Then, the dynamics of the system can be described exactly through a continuous-time Markov process with 2^*N*^ configurations [19], ranging from all patches being empty to all being occupied. In the absence of external input, the Markov process has only one absorbing state, the one in which the metapopulation is extinct and all patches are empty. Despite the fact that this is the ultimately absorbing state, the dynamics are dominated for a long time by a quasi-stable state, in which a characteristic proportion of patches are occupied. This quasi-stable state is the focus of our work.

Because such large Markov processes are difficult to treat analytically, researchers have sought ways to approximate the dynamics using simpler models. Interestingly, the same class of models originated in two distinct scientific communities: one interested in metapopulations and conservation biology [3, 20, 21], the other interested in the spread of infectious diseases in a social network [19, 22, 23]. In fact, a metapopulation model can be turned into a Susceptible-Infected-Susceptible (SIS) model by relabeling “patches” as “individuals”, “dispersal” as “infection”, and “extinction” as “recovery”. Though the two scientific communities have opposite goals (preservation of metapopulations versus eradication of diseases), their models are identical, so results in one area directly translate into the other.

In particular, both communities studied an approximation in which the system is modeled by *N* differential equations, each tracking the probability that a certain patch is occupied/individual is infected. Besides simplifying calculations, this approximation has the added benefit that the quasi-stable state of the Markov process becomes the steady-state of the differential equation model. Here we follow the formulation found in the SIS literature [23, 24], which might be new to readers more familiar with the metapopulation literature. Doing so highlights the critical step made to approximate the dynamics as well as the strong connection between metapopulation dynamics and disease spread.

We have *N* patches/individuals *S*_*i*_(*t*), each taking value 1 or 0 (i.e., each being a Bernoulli random variable) at time *t*, depending on whether the given patch is occupied / individual is infected. Let *δ*_*i*_ be the probability per unit time of patch *i* going extinct / individual *i* recovering. Let *D*_*ij*_ be the probability per unit time that, given that patch *j* is occupied while patch *i* is empty, patch *j* is going to colonize patch *i* (individual *j* is infected while individual *i* is uninfected, individual *j* is going to infect individual *i*). Assuming that both colonization and extinction (infection and recovery) are Poisson processes, one can write a system of ordinary differential equations tracking the dynamics of the expectations 𝔼[*S*_*i*_(*t*)] in time:

$$\begin{array}{c} \frac{d𝔼[S_{i}\left(t\right)]}{dt}=-\delta_{i}𝔼\left[S_{i}\left(t\right)\right]+\sum_{j\neq i}D_{ij}𝔼\left[S_{j}\left(t\right)\right]-\sum_{j\neq i}D_{ij}𝔼\left[S_{i}\left(t\right)S_{j}\left(t\right)\right] \end{array}$$ (1)

Assuming independence, one can write 𝔼[*S*_*i*_(*t*)*S*_*j*_(*t*)] = 𝔼[*S*_*i*_(*t*)]𝔼[*S*_*j*_(*t*)], thus obtaining the Hanski-Ovaskainen model in metapopulation theory [3, 20, 21] or the so-called NIMFA model in the SIS literature [23]:

$$\begin{array}{c} \frac{d𝔼[S_{i}\left(t\right)]}{dt}=-\delta_{i}𝔼\left[S_{i}\left(t\right)\right]+\left(1-𝔼\left[S_{i}\left(t\right)\right]\right)\sum_{j\neq i}D_{ij}𝔼\left[S_{j}\left(t\right)\right] \end{array}$$ (2)

In general, the variables *S*_*i*_(*t*) and *S*_*j*_(*t*) will be correlated. However, it has recently been proven that if extinction/cure and colonization/transmission are Poisson processes, the correlation can only be positive [25]. This means that the above equation necessarily overestimates the growth of the probability that patches are occupied/individuals are infected. In our setting, this turns out to guarantee that our estimate of the persistence threshold of the metapopulation is always conservative [19]. Moreover, in the disease literature a treatment of the second-order approximation (accounting for pairwise correlations, but foregoing third-order ones) has appeared [24].

### The Hanski-Ovaskainen model

Here we generalize the model proposed by Hanski & Ovaskainen [3] starting from Eq (2). The *N* patches of suitable habitat are positioned in a *d*-dimensional landscape [26]. Each patch is characterized by a position *x*_*i*_ (a vector of length *d*), and by a patch value *A*_*i*_, expressing for example the carrying capacity of the patch. The extinction rate in patch *i* is given by a general extinction rate *δ* (a property of the species in question and of the landscape as a whole), whose effect is mitigated in patches of high value: *δ*_*i*_ = *δ*/*A*_*i*_. Individuals can disperse between patches with a rate that depends on the distance between the two patches, a dispersal kernel function, and the value of the patch from which individuals disperse: *D*_*ij*_ = *A*_*j*_ *f*(|*x*_*i*_−*x*_*j*_|/*ξ*), where *f* is the dispersal kernel function, |*x*_*i*_−*x*_*j*_| is the Euclidean distance between patches *i* and *j*, and *ξ* is the typical dispersal length for individuals of the species of interest.

Taking Eq (2), writing *p*_*i*_(*t*) instead of 𝔼[*S*_*i*_(*t*)], and substituting in the above expressions, we obtain the model:

$$\begin{array}{c} \frac{dp_{i}\left(t\right)}{dt}=\left(1-p_{i}\left(t\right)\right)\sum_{j\neq i}f(\frac{|x_{i}-x_{j}|}{\xi })A_{j}p_{j}\left(t\right)-\frac{\delta }{A_{i}}p_{i}\left(t\right)\;. \end{array}$$ (3)

where *p*_*i*_(*t*) is the probability of finding the species of interest in patch *i* at time *t*, and the contribution of each patch *j* to the probability of finding the species in *i* is weighted by the dispersal kernel *f*, the patch value *A*_*j*_ and the probability of occurrence in patch *j*, *p*_*j*_(*t*). The extinction rate *δ* is mitigated in patches of high value.

This system of equations has at least one equilibrium *p*, and the metapopulation is persistent when ∑_*i*_ *p*_*i*_ > 0. Interestingly, if the equilibrium *p* is strictly positive, then it is also stable [20]. Although one cannot analytically solve for the equilibrium, all the information needed to calculate the persistence of the system is enclosed in the so-called dispersal matrix *M*, whose diagonal entries are zero, and off-diagonal are defined as *M*_*ij*_ = *M*_*ji*_ = *A*_*i*_ *A*_*j*_ *f*(|*x*_*i*_−*x*_*j*_|/*ξ*).

The leading eigenvalue of *M*, *λ* (“metapopulation capacity” [3]), determines the persistence of the metapopulation. Specifically, the metapopulation can be persistent if and only if *λ* > *δ* [3, 21]. As such, probing the dependence of *λ* on different factors is crucial to predicting persistence. If the leading eigenvalue determines persistence, the corresponding eigenvector *w*_*i*_ quantifies the relative importance of patches [20, 27] and, when *δ* ≈ *λ*, it is related to the stationary solution *p* itself [20] (S1 Text).

Habitat destruction (i.e., the removal of patches) lowers the metapopulation capacity. Mathematically, patch removal is carried out removing a row and the corresponding column from the matrix *M*. The removal of a single patch has a negative effect on *λ*, but the magnitude of the effect depends on patch identity. The relationship between the eigenvalue and the eigenvector [27] quantifies the effect of the removal of patch *i* on the metapopulation capacity:

$$\begin{array}{c} \frac{\lambda -\lambda_{i}^{-}}{\lambda }=\frac{\Delta \lambda_{i}}{\lambda }\approx w_{i}^{2}\;, \end{array}$$ (4)

where *λ* is the metapopulation capacity before removal, $\lambda_{i}^{-}$ is the metapopulation capacity after the removal of patch *i*, and *w* is the normalized ($\sum_{i}w_{i}^{2}=1$) dominant eigenvector of *M* (before removal). Thus, the components of the eigenvector quantify patch importance, as typically found in the realm of complex networks when measuring eigenvector centrality [28].

### Random fragmented landscapes

Here we study the model in Eq (3) when patches are randomly distributed in the landscape. We take a large number of patches *N* randomly positioned in a *d*-dimensional cube by sampling their positions from a uniform distribution. The cube has sides of length *L* (with *L* ≫ *ξ* to avoid dealing with edge effects).

Although in principle patch values could be correlated (e.g., high-value patches being close to each other), for simplicity we sample the *A*_*i*_ independently from a distribution with mean one and variance *σ* ^2^.

We examine different kernel functions (Hanski & Ovaskainen considered only one), and we stress that, unless specified, our results hold for any kernel function, including those that are not monotonically decreasing (S1 Text). For illustration, we use the Exponential, Gaussian and Rectangular kernel functions (Fig 1).

## Results

### Will a metapopulation in a random landscape be persistent?

Knowing the number of dimensions (*d*) and size (*L*) of the landscape, the number of patches *N*, the dispersal kernel *f*(|*x*_*i*_−*x*_*j*_|/*ξ*), and the distribution of the values of the patches (*σ* ^2^), we want to approximate *λ*, the metapopulation capacity.

The matrix *M* is nonnegative (*M*_*ij*_ ≥ 0) and symmetric (*M*_*ij*_ = *M*_*ji*_), therefore *λ* is bounded by the average row sum from below [29].

Take the patch values to be one for all patches, and assume a rectangular kernel (Random Geometric Graph). Then, the average row sum is simply the average number of neighbors each patch has (the average degree of the network). When using another kernel (Euclidean Random Matrix), the row sum can be interpreted as the average number of “effective neighbors”, *n*_*e*_, meaning that patches that are closer contribute more to the sum than those that are far away. As such, when all patches have value one, *λ* ≥ *n*_*e*_. When the patch values are sampled from a distribution with mean one and variance *σ* ^2^, assuming *N* large, we obtain *λ* ≥ *n*_*e*_(1+*σ* ^2^) (S1 Text). This provides a conservative criterion for metapopulation persistence:

$$\begin{array}{c} n_{e}\left(1+\sigma^{2}\right)>\delta \;. \end{array}$$ (5)

Next, we want to approximate *n*_*e*_ for different parameterizations. In fact, *n*_*e*_ is influenced by the dispersal kernel, the dispersal length, the number of dimensions, the number of patches, and the size of the landscape. The formula, in the limit *L* ≫ *ξ*, reads

$$\begin{array}{c} n_{e}\approx \frac{N}{L^{d}}\;G_{f}\left(d\right)\xi^{d} \end{array}$$ (6)

(S1 Text), where the first term measures the patch density, obtained by dividing the number of patches *N* by the volume *L* ^*d*^ (area if *d* = 2, or length if *d* = 1), while the second term (*G*_*f*_(*d*)*ξ* ^*d*^) measures the typical volume accessible via dispersal from a patch. Their product *n*_*e*_ represents therefore the typical number of patches accessible via dispersal. The numerical factor *G*_*f*_(*d*) depends on the functional form of the dispersal kernel and on *d*. For example, *G*_*f*_(*d*) = (2*π*)^*d*/2^ for the Gaussian kernel (S1 Text). Note that although we kept *ξ* and *L* distinct, we could have measured one in units of the other, as what matters for the metapopulation capacity is their ratio.

**Fig 2:**
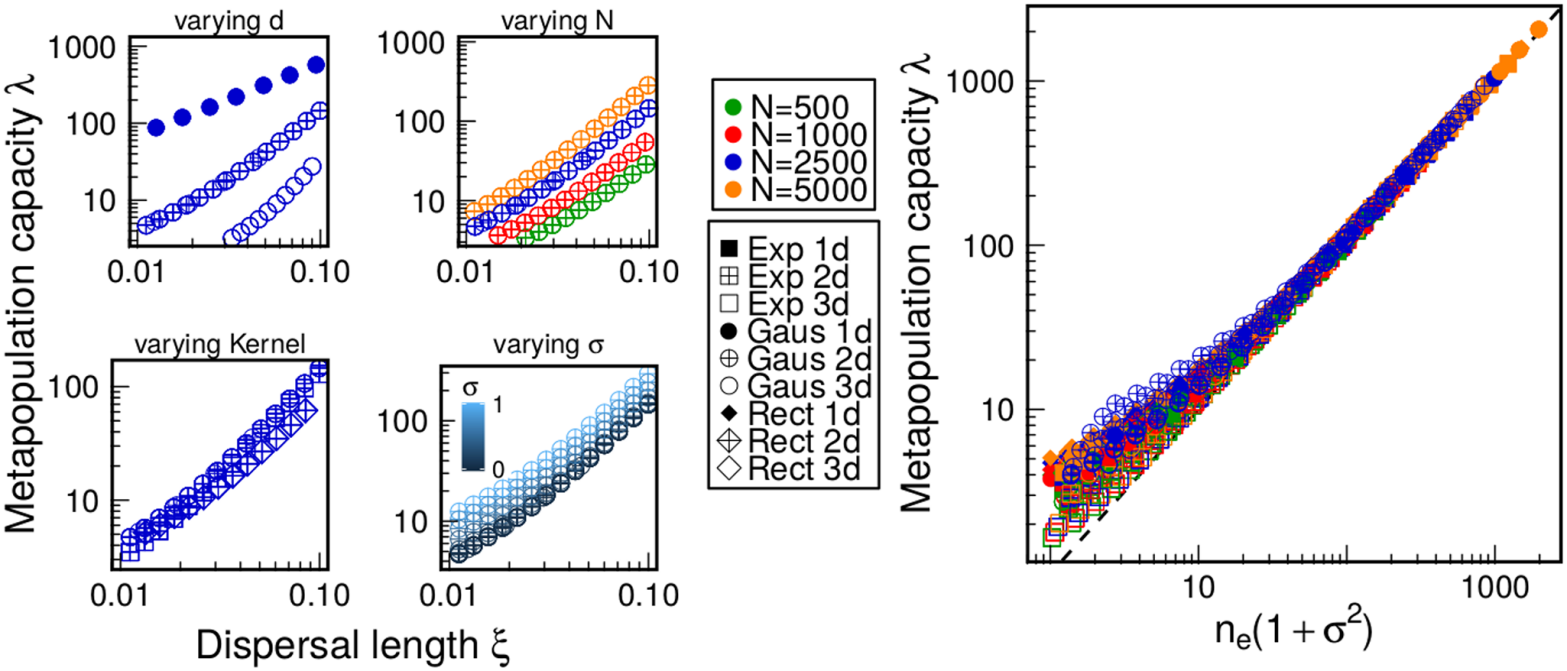
Dependence of the metapopulation capacity on various factors. The four panels on the left show the metapopulation capacity *λ* vs. the dispersal length *ξ*. *λ* depends on the number of dimensions *d* (1, 2, 3), the number of patches *N* (500, 1000, 2500, 5000), the dispersal kernel *f* (Gaussian, Exponential and Rectangular), and the heterogeneity of patch value *σ* (varied between 0 and 1). Different symbols correspond to different combinations of *f* and *d*, while different colors correspond to different values of *N*. The color scale from blue to light blue refers to different values of *σ*. The right panel shows the collapsing of the various curves into a single one. The dependence on all the parameters (namely *d*, *N*, *ξ*, *f* and *σ*) is amalgamated in *n*_*e*_(1+*σ* ^2^), where *n*_*e*_ is a simple function of all the parameters (computed via Monte Carlo integration, see S1 Text). The region of largest discrepancy (small *n*_*e*_(1+*σ* ^2^)) corresponds to the localized case, where only few patches contribute to the persistence of the metapopulation (S1 Text). Without loss of generality, we set *L* = 1 in all simulations.

Fig 2 shows that *λ* depends on all the factors that are needed to estimate *n*_*e*_: the number of patches *N*, the kernel *f*, the dispersal length *ξ*, and the number of dimensions *d*. Moreover, *λ* depends on the probability distribution function for the value of the patches. However, when plotting *λ* versus *n*_*e*_(1+*σ* ^2^), where *σ* ^2^ is the variance of the distribution, all curves collapse into the same one, meaning that two very different fragmented landscapes, or with different distribution of patch values but the same value of *n*_*e*_(1+*σ* ^2^), have approximately the same metapopulation capacity (S1 Text).

### A regular arrangement of the patches decreases persistence

In the previous section, we derived a persistence criterion for the case in which the patches are randomly distributed in a *d*-dimensional cube. This random arrangement is among the most “disordered” possible. We now examine how a more regular arrangement of patches would affect persistence.

We arrange the patches in a regular grid spanning the *d*-dimensional cube. We then perturb this regular arrangement by jiggling the patch positions described by the parameter *η*. When *η* = 0 the patches are perfectly arranged in a grid, and when *η* = 1, the patches are distributed at random (S1 Text). Tuning *η*, we can explore the effect of intermediate states between the fully regular and fully random.

If the patches are arranged in a regular grid, *M* has a particularly simple structure. In fact, if the space had no edges (as in a *d*-dimensional torus), all the rows of *M* would sum to exactly the same constant *λ*_grid_, the leading eigenvalue of *M*. For a sufficiently large number of patches, approximately the same is found when the space is bounded. As mentioned before, *λ* is larger than or equal to the average row sum of *M*, but since here all rows have the same sum, we obtain equality (S1 Text). This means that *λ*_grid_ is always lower than the corresponding *λ*_rand_, found for a system with the exact same average row sum, but with random patch positions. Simulations show that—all other things being equal—increasing *η* always increases the leading eigenvalue (S1 Text), so that the likelihood of persistence is increased by disordered patch arrangements.

**Fig 3:**
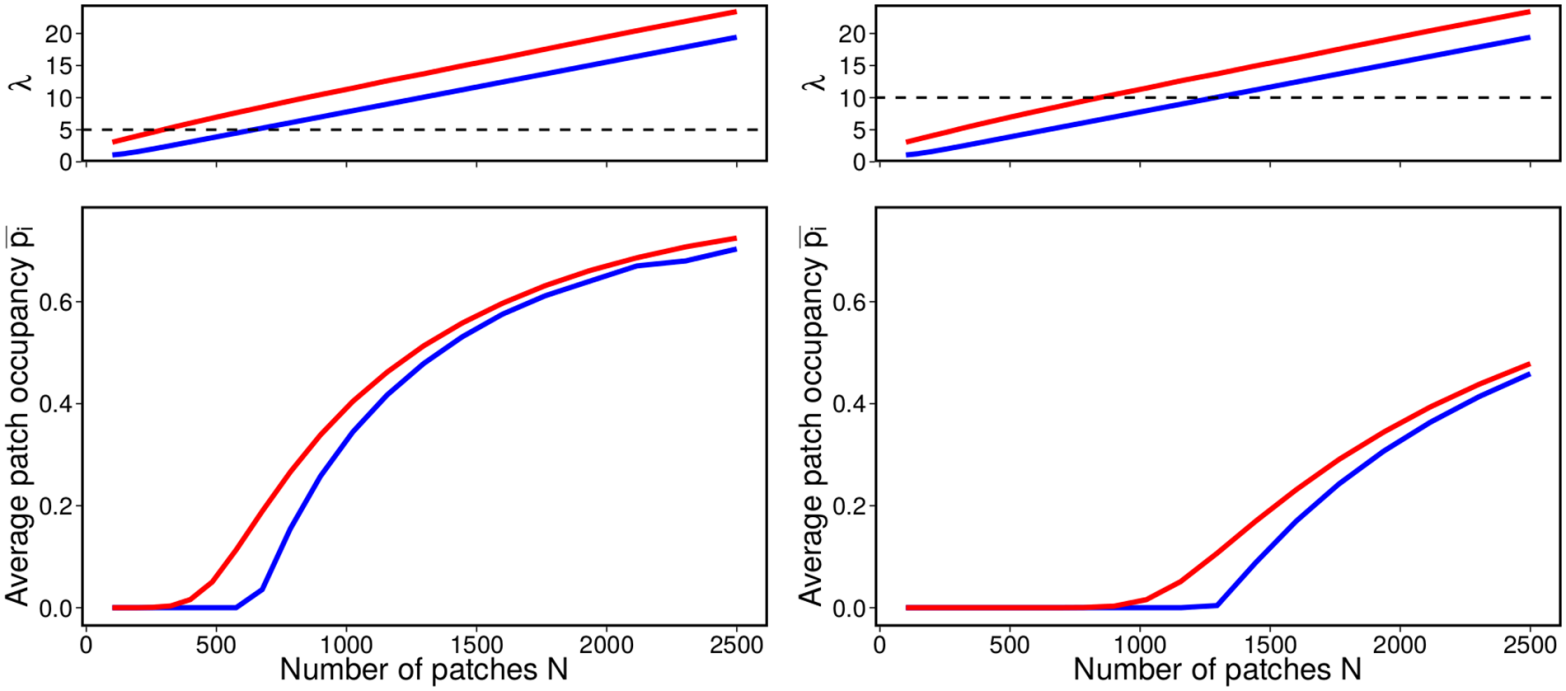
Effect of patch arrangement on persistence: grid vs. random. Blue lines refer to patches arranged in a perfect grid spanning the landscape, while red lines refer to random positions. The metapopulation capacity vs. the number of patches *N* (top). The right and left columns refer to two values of *δ* (dashed lines), yielding a different profile for the average number of occupied patches $\bar{p_{i}}=\sum_{i}p_{i}/N$ vs. *N* (bottom). In both cases, the eigenvalue scales linearly with the number of patches *N* (see Eq 6), but it is always larger for the case of randomly arranged patches. Similarly, a random arrangement leads to a higher average occupancy. The simulations are performed for a Gaussian kernel, *d* = 2, *L* = 1, *ξ* = 0.05.

Both in the grid and in the random arrangements, the metapopulation capacity grows linearly with the number of patches (Fig 3; see also Eq 6), and we always find *λ*_grid_ < *λ*_rand_. Similarly, when we take the average patch occupancy $\bar{p_{i}}$, measuring the proportion of occupied patches, we find that randomly distributing the patches leads to a higher (when *λ*_rand_ > *δ*) or equal (when both *λ*_rand_ and *λ*_grid_ are lower than *δ*, and thus $\bar{p_{i}}=0$) proportion of occupied patches (Fig 3).

So far, we have considered monotonically decreasing dispersal kernels, in which the proverbial seed never falls far from the tree. This type of kernel was the first being studied [3], and is frequently encountered in empirical analyses [30]. However, trees have strategies to disperse their seeds (e.g., fruits for seed-dispersers, wind dispersal), while seeds falling close to the tree could be disproportionally consumed by predators (Janzen-Connell hypothesis), giving rise to more complex shapes for the dispersal kernel [31].

In the case of monotonically decreasing dispersal kernels (such as exponential or Gaussian ones), it is possible to demonstrate analytically that *λ*_grid_ < *λ*_rand_ (S1 Text). In the case of non-monotonically decreasing, hump-shaped dispersal kernels, the same proof does not hold in general. Despite this however, numerical simulations (S1 Text) show that, even if the kernel is fine-tuned to peak around the nearest neighbor in a grid, the metapopulation capacity is still larger for a random assembly of patches than a regular arrangement. This is a counterintuitive result, since in principle we can always imagine a kernel which is zero everywhere except in an infinitely narrow band around the nearest neighbor distance. Then the slightest perturbation away from a perfect grid structure would immediately reduce the metapopulation capacity to zero. Based on this extreme case scenario, one may justifiably think that slightly relaxing the assumption of an infinitely sharp peak in the kernel will still result in a grid arrangement being more beneficial than a random one. Our results show that this is not so: even a very slight smearing of the kernel from the extreme-peaked case leads in practice to a random arrangement of patches having a higher metapopulation capacity than the grid arrangement.

### Spatial localization in metapopulations close to extinction

In network theory, the leading eigenvector measures nodes’ importance, with applications in many different areas [32–34], including metapopulation theory [3, 20, 27].

Given that the leading eigenvalue of *M* determines the persistence of the metapopulation, naturally its value decreases when patches are removed, and it can be shown [27] that when patch *i* is removed, the relative change of the eigenvalue is approximately equal to $w_{i}^{2}$, where *w*_*i*_ is the *i*-th component of the eigenvector *w* (Eq 4).

**Fig 4:**
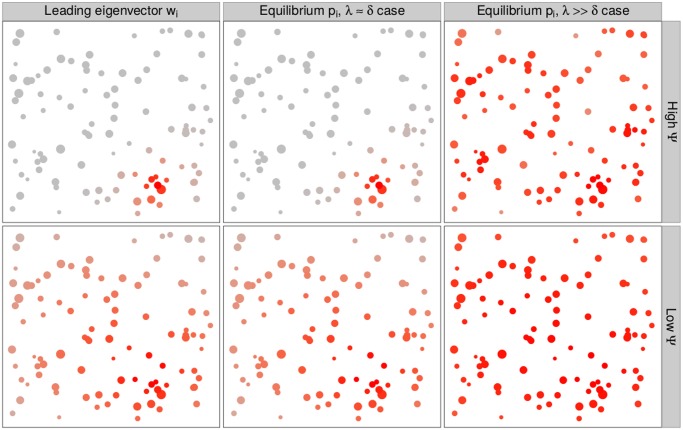
Spatial localization of metapopulations close to extinction. The metapopulation can persist in two different regimes: for small *n*_*e*_(1+*σ* ^2^), and therefore small *λ*, the leading eigenvector (i.e., that associated with *λ*) is highly heterogeneous (high Ψ, top left), while for large *n*_*e*_(1+*σ* ^2^), all patches have roughly the same eigenvector component (low Ψ, bottom left). When the metapopulation is close to extinction (middle column, *λ* ≈ *δ*), the equilibrium values *p*_*i*_ are well approximated by the eigenvector component *w*_*i*_. When the eigenvector is heterogeneous, the metapopulation is maintained by few patches with high probability of persistence (those with high *w*_*i*_). The interesting feature is that these patches are spatially localized, so that a small region of the landscape contributes disproportionately to persistence. This is not the case when *λ* ≫ *δ*, in which case multiple eigenvectors influence *p*, resulting in an almost uniform distribution of the *p*_*i*_ (right column).

If we assume that every patch is equally connected to every other patch, as in Levins’s model [2], all the patches have the same importance. Our model predicts a radically different scenario, in which the importance of the different patches can be extremely heterogeneous (Fig 4).

To quantify heterogeneity, we introduce Ψ, a measure related to the variance of $w_{i}^{2}$ (S1 Text). Ψ is zero if all the patches have the same importance, and is one if only a single patch contributes to the leading eigenvector, i.e., all but one component of the eigenvector are zero. Ψ depends both on *n*_*e*_ and *σ* ^2^. As the variance of patch values *σ* ^2^ increases, Ψ increases (S1 Text). The dependence on *n*_*e*_ is more subtle and interesting: for large values of *n*_*e*_, Ψ is close to zero, while, as *n*_*e*_ decreases, Ψ remains close to zero up to a critical value, at which point it suddenly increases (S1 Text). This critical value corresponds to a transition from a system where all patches have more or less the same importance for metapopulation persistence to one where a few patches, localized in space, contribute disproportionately to the eigenvector (Fig 4).

We emphasize that this result holds for any value of the extinction rate *δ*. In addition, in the *λ* ≈ *δ* limit, i.e., when the metapopulation is close to the extinction threshold, the eigenvector is also related to the stationary solution *p* (S1 Text). Close to the extinction threshold the persistent patches are spatially localized, i.e., the patches with high likelihood of persistence are all close in space.

In the previous section, we showed that the leading eigenvalue of *M* for a landscape in which patches are arranged in a grid is always smaller than that obtained for randomly distributed patches. A similar pattern holds also for the eigenvector: the variance of the eigenvector components of a perfect grid is always zero (S1 Text), as all the patches have the same importance. However, when we disturb the arrangement, we find that, for *n*_*e*_(1+*σ* ^2^) small, the variance in the importance of patches Ψ increases rapidly (S1 Text). The important patches, i.e., the patches associated with a large eigenvector component, are not uniformly distributed in space, but rather they are spatially clustered (S1 Text). As such, not only the eigenvector is localized (large Ψ), but the localization is spatial, with all the important patches enclosed in a small region of the landscape (Fig 4).

## Discussion

By modeling fragmented landscapes as networks [4, 6] in which the nodes are patches and the weighted edges represent dispersal, we have shown that metapopulation persistence can be studied analytically for the case in which patches are randomly distributed in the landscape, and patch values are independently sampled from some distribution.

The derivation highlights that a few key quantities determine the metapopulation capacity: the density of the patches, with denser landscapes yielding a higher probability of persistence; the shape of the dispersal kernel; the number of dimensions; and the variability in patch value, with higher variance being beneficial.

Our analysis provides a null model for metapopulation persistence. For a given empirical landscape, in which the patches positions are not necessarily described by a uniform distribution and patch values are not independent of patch position [7, 26], the effect of these features on persistence can be disentangled from the effects of other factors by contrasting the metapopulation capacity of the empirical landscape with what is expected according to our framework.

Interestingly, we found that even a very small amount of disorder and variation are beneficial for persistence. First, the variance in patch values has a very strong positive effect on the metapopulation capacity, meaning that highly heterogeneous patch values yield higher *λ* than homogeneous ones. Second, we found that more “disordered” arrangements of the patches increase both the metapopulation capacity and the expected proportion of occupied patches. This is especially relevant when metapopulations are close to extinction, as localization becomes key for maintaining the metapopulation viable, albeit in a spatially confined region. Note that these analytic results shed light on previous simulations suggesting that unequal spacing between the patches is beneficial for populations close to extinction [18], and are consistent with what was found when considering stochastic dynamics [35].

The development in the study of metapopulation models is paralleled by that of contact processes in infectious diseases. Our results could find applications in the case of diseases spreading on a “geographic” network (e.g., agricultural pests). Similarly, the fact that variance in patch value increases the metapopulation capacity would suggest that adding individual variability to the recovery time and infectiousness would lead to faster transmission and an increased chance of epidemic outbreaks.

Although we have examined the case of randomly distributed patches using a uniform distribution, the same approach holds when the distribution is not uniform [26]. For example, riparian plants in a landscape crossed by a river will be concentrated in the vicinity of the water, leading to a non-uniform distribution. Fortunately, in the limit of many patches, any distribution can be taken into account, by evaluating analytically or numerically the integral defining *n*_*e*_ in the general case (S1 Text). Similarly, we accounted for an integer number of dimensions, but a fractional *d* (e.g., in a fractal-like river basin) would not alter the framework. Finally, we assumed that species can disperse equally in all directions, with no preference. When there is a clear preferential direction of dispersal (e.g., wind-dispersal of seeds, or fish larvae dispersing in a river), the theory of Euclidean Random Matrices can be extended to account for this lack of symmetry [36]. There are indeed several ways to generalize this work. One can consider a scenario where extintion and colonization have a different dependence on patch values or include a dependence on the latter in the dispersal kernel. These complications can all be viewed as generalizations of our approach, where new correlations are introduced between the elements of the random matrix.

The derivation of a criterion for metapopulation persistence bears a striking resemblance to the derivation of stability criteria for large ecological communities [37, 38], as in both cases the use of random matrix theory led to the identification of the few basic parameters responsible for the large-scale behavior of the systems. The advantage of this approach is that, once the modeling of the matrices is in place, the derivation of the results requires only elementary algebra. Random matrix theory is currently experiencing an impressive growth [39], greatly expanding the potential for biological applications.

## Supporting Information

S1 Text Supplementary Methods and Results. Background on Species Occupancy Models (SPOMs); approximations of the stationary state; derivation of the persistence criterion; supplementary results on nonmonotonic kernels.
(PDF)
