# Supplementary material for: Metapopulation Persistence in Random Fragmented Landscapes
Source: PLoS Comput Biol. 2015 May 20;11(5):e1004251. doi: 10.1371/journal.pcbi.1004251 (PMC4439033; doi:10.1371/journal.pcbi.1004251)
Supplement: S1 Text — Background on Species Occupancy Models (SPOMs); approximations of the stationary state; derivation of the persistence criterion; supplementary results on nonmonotonic kernels. (PDF) [file pcbi.1004251.s001.pdf]

Metapopulation persistence in random fragmented landscapes Supporting Information

Jacopo Grilli[1], György Barabás[2], Stefano Allesina[2,3,*]

1 Department of Physics and Astronomy 'G. Galilei', Università di Padova, Via Marzolo 8, 35131 Padova, Italy.
2 Department of Ecology & Evolution, University of Chicago, 1101 E 57th St, Chicago, IL 60637, USA.
3 Computation Institute, University of Chicago.

*** sallesina@uchicago.edu**

S1 Text

1 Stochastic patch occupancy models

The starting point for stochastic patch occupancy models (SPOMs, [21]) is Levins's model [2]. The model assumes a large number of identical patches of suitable habitat that may or may not be occupied by a local population. The proportion of patches $p(t)$ that are occupied at time t is governed by the equation

$$\frac{\mathrm{d}p(t)}{\mathrm{d}t} = cp(t)\,(1-p(t)) - ep(t), \tag{1}$$

where c is the colonization rate and e is the extinction rate. The metapopulation is persistent whenever $e/c < 1$, i.e., the colonization rate exceeds the extinction rate.

This model is spatially implicit: individual patches and local population persistence probabilities are not modeled explicitly. To make the model spatially explicit, one should specify the location of the patches in the landscape. The geometry of the landscape will then dictate the rate at which individuals in a patch colonize other patches, with colonization rates typically decreasing with the distance between the patches. Moreover, patches can differ in size or suitability such that the dynamics of a local population depends on the patch "value".

Hanski & Ovaskainen [3,20,21] proposed a model in which the colonization and extinction rates depend both on the particular patch under consideration and the occupancy state of all the other patches:

$$\frac{\mathrm{d}p_i(t)}{\mathrm{d}t} = (1-p_i(t))\,C_i - p_i(t)E_i, \tag{2}$$

where $p_i(t)$ is the probability that patch i is occupied at time t, and $C_i = C_i\,(p_1(t),\ldots,p_N(t))$ and $E_i = E_i\,(p_1(t),\ldots,p_N(t))$ are the colonization and extinction rates of patch i as a function of the current distribution of the population over the landscape (N is the total number of patches).

The model also incorporates the notion of patch "value" (e.g., a function of the patch size and the density of available resources), which measures the probability of incidence on a given patch without any immigration from other patches, and can

therefore be interpreted as the carrying capacity of the patch. The colonization rates are assumed to be directly proportional to this value, while the extinction rates are assumed to be inversely proportional to it. Denoting the value of patch i by A_i, the extinction rate in patch i is $E_i = \delta/A_i$, where δ is the extinction rate per unit patch value. Without loss of generality we assume that, up to a rescaling of δ, the mean patch value (averaged over all the N patches) is one, i.e., $\sum_i A_i/N = 1$.

The colonization rate of patch i is composed of the contribution of all other patches, with the contribution of each patch being a function of the distance between the patches. This function is called the *dispersal kernel* and is denoted $f\left(|x_i - x_j|/\xi\right)$, where x_i is the spatial position of patch i, $|x_i - x_j|$ is the Euclidean distance between patches i and j, and ξ is the *dispersal distance*, i.e., the characteristic distance the population can traverse in a unit of time. The colonization rate is therefore $C_i = \sum_{j\neq i}^{N} f\left(|x_i - x_j|/\xi\right) A_j p_j$. Hanski & Ovaskainen [2] studied the special case of an exponential dispersal kernel $f\left(|x_i - x_j|/\xi\right) = \exp\left(-|x_i - x_j|/\xi\right)$, but for a general kernel, we have:

$$\frac{\mathrm{d}p_i(t)}{\mathrm{d}t} = \sum_{j\neq i}^{N} \left(1 - p_i(t)\right) f\left(\frac{|x_i - x_j|}{\xi}\right) A_j p_j(t) - \frac{\delta}{A_i} p_i(t). \tag{3}$$

Introducing the matrix M with the definition

$$M_{ij} = \begin{cases} f\left(\frac{|x_i - x_j|}{\xi}\right) A_i A_j, & i \neq j, \\ 0, & i = j, \end{cases} \tag{4}$$

Hanski & Ovaskainen [2] derived a simple persistence rule: the metapopulation persists as long as the extinction rate $\delta < \lambda$, where λ is the leading (largest, rightmost) eigenvalue of M. Thus, the criterion for metapopulation persistence has the same form found in Levins's model, with the leading eigenvalue λ (dubbed the "metapopulation capacity" [2]) playing a key role for persistence.

2 The dispersal matrix

The dispersal matrix M (Eq. 4) has special properties as long as the dispersal rate depends only on the distance between patches, which we will assume throughout. In this case M is symmetric ($M_{ij} = M_{ji}$) and nonnegative ($M_{ij} \geq 0$). Symmetry implies that all the eigenvalues of M are real. Then, as long as M is irreducible (a condition typically met by the matrices studied here), M will have a unique largest eigenvalue [40, 41], which is the leading eigenvalue λ referred to above. Moreover, the eigenvector associated with this eigenvalue can be chosen with strictly positive components. Note that, since we are interested in the largest eigenvalue overall as opposed to the largest in absolute value, primitivity of M is not required.

Moreover, using the symmetry of M, one can derive a lower bound for the magnitude of λ. Since by definition

$$\lambda = \max_{\theta_i \neq 0} \left(\frac{\sum_{i=1}^{N} \sum_{j=1}^{N} \theta_i M_{ij} \theta_j}{\sum_{k=1}^{N} \theta_k^2} \right), \tag{5}$$

we may arbitrarily set $\theta_i = 1$ for every i to obtain

$$\lambda \geq \frac{1}{N} \sum_{i=1}^{N} \sum_{j=1}^{N} M_{ij} \tag{6}$$

(e.g., [29]). This sum can be interpreted as the arithmetic mean of the row sums of the matrix M. Therefore, the average row sum provides a lower bound for the leading eigenvalue of symmetric matrices.

This approximation is in fact a special case of the more general formula

$$\lambda \geq \frac{\sum_{i=1}^{N} \sum_{j=1}^{N} (M^q)_{ij}}{\sum_{i=1}^{N} \sum_{j=1}^{N} (M^{q-1})_{ij}} \tag{7}$$

where q is a positive integer. Note that for $q = 1$ we recover Eq. (6).

3 The stationary state

The metapopulation capacity λ provides a persistence condition, but does not contain information on the structure of the metapopulation. In order to study metapopulation structure, we need to know the equilibrium patch occupancy probabilities p_i for each patch as a starting point. In principle, this amounts to solving Eq. (3) at equilibrium, i.e., when $\mathrm{d}p_i(t)/\mathrm{d}t = 0$:

$$\sum_{j \neq i}^{N} (1 - p_i) f\left(\frac{|x_i - x_j|}{\xi}\right) A_j p_j = \frac{\delta}{A_i} p_i. \tag{8}$$

For brevity, we write p_i for the probability that patch i is occupied, evaluated at equilibrium. Multiplying both sides by A_i and using Eq. (4), we get

$$\sum_{j=1}^{N} (1 - p_i) M_{ij} p_j = \delta p_i. \tag{9}$$

Unfortunately, Eq. (9) cannot be solved analytically in the general case. It is possible however to derive approximate expressions for the p_i under various limiting scenarios. One of these limits is the strong persistence limit, when $\lambda \gg \delta$; as we will show, in this case we have

$$p_i \approx 1 - \left(\frac{\delta}{\sum_{l=1}^{N} M_{il}}\right). \tag{10}$$

The other is the $\lambda \approx \delta$ limit, i.e., when the metapopulation is close to the extinction threshold. In this case

$$p_i \approx \frac{\lambda - \delta}{\lambda} \frac{w_i}{\sum_{j=1}^{N} w_j^3} \tag{11}$$

holds [20], where w_i is the ith component of the leading eigenvector of M (i.e., the eigenvector associated with λ). The quality of this approximation is shown in Fig. S1. In Section 10 we explicitly derive the two approximations.

From a conservation standpoint, the goal is to have metapopulations which persist robustly, and are therefore well described by the first approximation scheme ($\lambda \gg \delta$). As we have seen, the equilibrium patch occupancy probabilities are then given by Eq. (10), and only depend on δ and the matrix M. The general structure of the metapopulation distribution, however, is already evident just from the row sum structure of M. Despite the fact that Eq.(3) is a complicated object that is difficult to solve in general, much of its behavior can be deduced from knowledge of just this matrix.

The most interesting case, and unfortunately the one closer to reality for many populations, is the situation in which the metapopulation is close to extinction. For

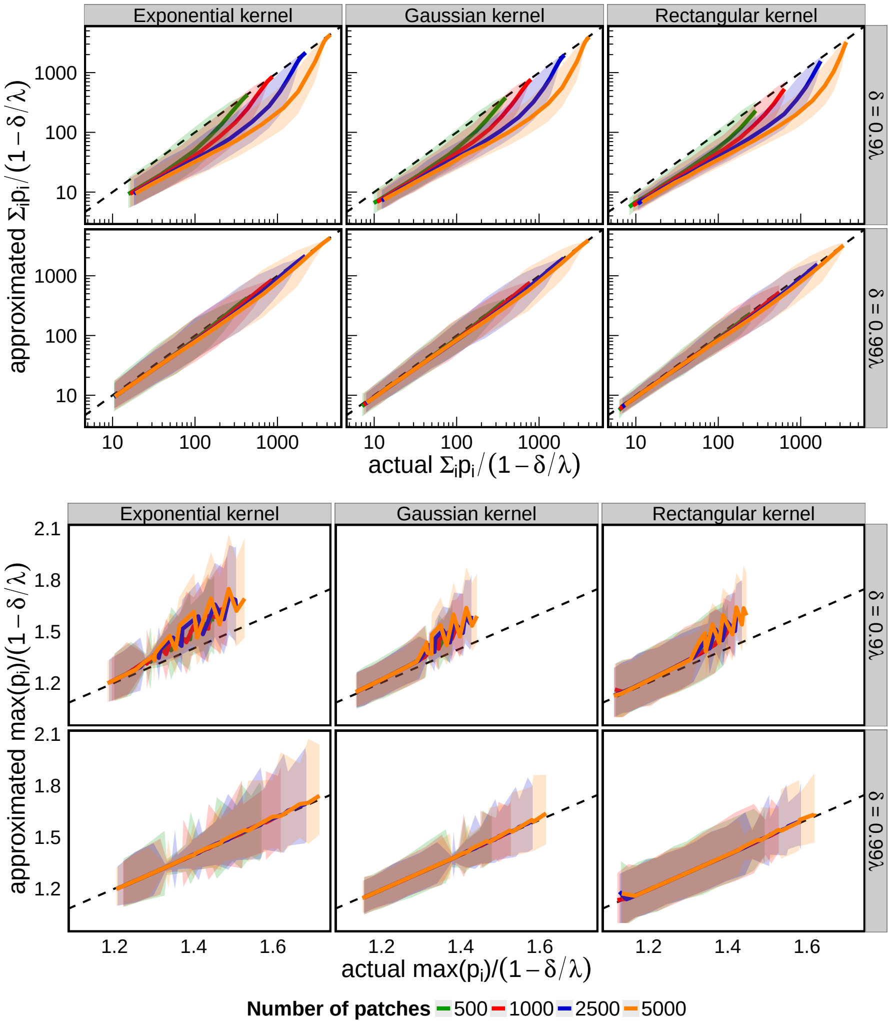

Fig. S1 The goodness of the approximate solution Eq. (11) when $\lambda \approx \delta$. Top panel: exact (x-axis, obtained by solving Eq. (9) numerically) vs. approximate (y-axis, obtained using Eq. (11)) solution for the sum of all persistence probabilities. Bottom panel: maximum probability of patch occupancy, $\max(p_i)$. In each panel, the top row is obtained for $\delta = 0.9\lambda$, and the second for populations even closer to extinction ($\delta = 0.99\lambda$). The columns represent different dispersal kernels (Section 5). The axes are scaled by $(1 - \delta/\lambda)^{-1}$ to make the visual comparison of the rows easier. The curves are obtained from simulations (1000 for each parameter combination) in which patches of identical value are randomly arranged on a square landscape of unit area, and the dispersal distance ξ is varied between 0 and 0.2 (see Section 5). The different colors stand for different numbers of patches. Shaded regions indicate the 95% of the resulting distribution. Clearly the approximation is quite good for $\delta = 0.9\lambda$ and improves dramatically for $\delta = 0.99\lambda$.

these metapopulations, the second approximation scheme ($\lambda \approx \delta$) is appropriate. In this case the equilibrium patch occupancies are well described by Eq. (11) (Fig. S1), an expression which—apart from the constant δ—depends only on the leading eigenvalue and eigenvector of M. Again, the interesting steady-state properties of Eq. (3) follow from M alone.

Having established that M governs not just the persistence condition [2], but also the equilibrium occupancies, in the remainder of this document we concentrate solely on M and its properties instead of the full dynamical equation Eq. (3).

4 Euclidean Random Matrices

Consider a region Ω in a d-dimensional Euclidean space (the landscape). Take a set of N points x_i randomly distributed over this region (the patches). Let $f(x_i, x_j)$ be an arbitrary function of any two points x_i, x_j in Ω. Then the matrix A with $A_{ij} = f(x_i, x_j)$ is called a *Euclidean Random Matrix* [15].

We analyze particular types of Euclidean Random Matrices: first, the points x_i are assumed to be uniformly distributed within the region Ω; second, we study dispersal kernels f that depend only on the distance between x_i and x_j: $f(x_i, x_j) = f(|x_i - x_j|/\xi)$, where ξ is a positive constant; third, the function f is assumed to be nonnegative. These assumptions arise from the interpretation of f as a dispersal kernel.

Sometimes, instead of working with the Euclidean Random Matrix A, we consider the matrix $B = A - f(0)I$, where I is the identity matrix. Since f is assumed to depend only on the distance between points, all diagonal elements of A are equal to $f(0)$. Therefore the only difference between A and B is that the latter has its diagonal entries set to zero. The effect of this is simply to shift the eigenvalues by a constant: if

$$Aw_\alpha = \lambda_\alpha w_\alpha \tag{12}$$

(where w_α, λ_α are the αth eigenvector and eigenvalue of A, respectively), then

$$Bw_\alpha = (A - f(0)I)\, w_\alpha = (\lambda_\alpha - f(0))\, w_\alpha. \tag{13}$$

meaning that the eigenvectors of B are the same as those of A, and that the eigenvalues of A are those of B shifted by $f(0)$.

Note that both A and B are symmetric nonnegative matrices, therefore the results of Section 2 hold. In particular, Eq. (6) provides a lower bound for their leading eigenvalues.

In the special case when f is equal to one for $|x_i - x_j|/\xi < 1$ and to zero otherwise (i.e. a rectangular dispersal kernel), the Euclidean Random Matrix this kernel generates is called a *Random Geometric Graph* [10]. We sometimes make use of Random Geometric Graphs, as they provide the most intuitive case of Euclidean Random Matrices, and they can be seen as the adjacency matrix of an undirected graph.

5 Metapopulation persistence

Whether a metapopulation can persist depends on the metapopulation capacity λ, which in turn depends on the dispersal kernel f, the number of spatial dimensions d, and the density of suitable habitat patches. Using the Euclidean Random Matrices introduced in Section 4, we derive a metapopulation persistence criterion for the case in which patches are uniformly distributed.

In this section we examine the case when all patches have equal value $A_i = 1$, and therefore the matrix M defined by Eq. (4) is a Euclidean Random Matrix, with diagonal

entries set to zero. Therefore the lower bound Eq. (6) can be used to approximate its leading eigenvalue. This bound is expressed as a double sum over the indices i and j which, in our case, means summing over the randomly distributed points in space:

$$\lambda \geq \frac{1}{N} \sum_{i=1}^{N} \sum_{j=1}^{N} M_{ij} = \frac{1}{N} \sum_{i=1}^{N} \sum_{j \neq i}^{N} f\left(\frac{|x_i - x_j|}{\xi}\right) \ . \tag{14}$$

As discussed in Section 2, the expression above can be seen as the average row sum of matrix M. When M is a Random Geometric Graph (Section 4), each entry M_{ij} is either equal to 1 if patch i can be reached from patch j, or to 0 otherwise. Then, the sum of all entries in row i is the number of patches that are reachable from the ith patch. In other words, it is the number of neighbors of patch i. The average row sum is then the average number of neighbors of the patches over the landscape.

For other dispersal kernels, we do not have such a clear distinction between patches that are reachable and those that are not. Still, the row sum of row i can be thought of as the *effective number of neighbors* of patch i, because patches that are more likely to be reached from i contribute more to the sum than those that are difficult to reach. Therefore, we interpret the average row sum of matrix M as the average effective number of neighbors, denoted n_e.

When the number of patches is large, i.e., $N \gg 1$, any summation over random points in a finite region Ω of space may be approximated by a continuous integral:

$$\frac{1}{N} \sum_{i=1}^{N} g(x_i) \xrightarrow{N \to \infty} \frac{1}{V_\Omega} \int g(x) \, \mathrm{d}x, \tag{15}$$

where g is an arbitrary function, V_Ω is the volume of the region Ω (or area for $d = 2$, length for $d = 1$), the integral runs over all of Ω, and it is understood that "dx" refers to integration for all d components of x. Hence, for $N \to \infty$

$$\frac{1}{N^2} \sum_{i=1}^{N} \sum_{j \neq i}^{N} f\left(\frac{|x_i - x_j|}{\xi}\right) \xrightarrow{N \to \infty} \frac{1}{V_\Omega^2} \iint f\left(\frac{|x - y|}{\xi}\right) \mathrm{d}x \, \mathrm{d}y. \tag{16}$$

The effective number of neighbors n_e can therefore be written, using the right hand side of Eq. (14), as

$$n_e = \frac{\rho}{V_\Omega} \iint f\left(\frac{|x - y|}{\xi}\right) \mathrm{d}x \, \mathrm{d}y \ , \tag{17}$$

where $\rho = N/V_\Omega$ is the density of patches over the landscape when $N \gg 1$. Then, from Eq. (14), we have $\lambda \geq n_e$. Since λ must be greater than δ for metapopulation persistence, this is a conservative estimate for the persistence condition: the metapopulation is expected to persist whenever the effective number of neighbors, estimated by the above integral, is larger than δ.

If we assume that the region Ω is the whole d-dimensional Euclidean space, the integration becomes especially simple. More precisely, we take the limit $N \to \infty$ and $V_\Omega \to \infty$ such that $\rho = N/V_\Omega$ remains finite. We then perform the change of variables $z = x - y$ to obtain

$$n_e = \rho \int f\left(\frac{|z|}{\xi}\right) \mathrm{d}z, \tag{18}$$

a much simpler integral than in Eq. (17).

Given the dispersal kernel, we can now derive the persistence condition. We illustrate how this is done using three different kernel forms assuming Ω is the whole

d-dimensional Euclidean space. The same calculation can be done for arbitrary other kernels in an analogous way.

- *Exponential kernel.* This kernel is given by

$$f\left(\frac{|x_i - x_j|}{\xi}\right) = \exp\left(-\frac{|x_i - x_j|}{\xi}\right), \tag{19}$$

where ξ is the dispersal distance. Let the points of Ω be denoted by x with $x_{(k)}$ being its kth coordinate ($k = 1 \ldots d$). Since we are integrating over the whole space, we can use Eq. (18), yielding

$$\begin{aligned} n_e =& \rho \int \exp\left(-\frac{\sqrt{\sum_{k=1}^{d} x_{(k)}^2}}{\xi}\right) \, \mathrm{d}x_{(1)} \cdots \mathrm{d}x_{(d)} \\ =& \frac{2\pi^{d/2}\Gamma(d)}{\Gamma(d/2)} \rho \xi^d \ , \end{aligned} \tag{20}$$

where Γ is the gamma function.

- *Gaussian kernel.* This is given by

$$f\left(\frac{|x_i - x_j|}{\xi}\right) = \exp\left(-\frac{|x_i - x_j|^2}{2\xi^2}\right) \tag{21}$$

with dispersal distance ξ. Again, we apply Eq. (18) to obtain

$$\begin{aligned} n_e =& \rho \int \exp\left(-\frac{\sum_{k=1}^{d} x_{(k)}^2}{2\xi^2}\right) \, \mathrm{d}x_{(1)} \cdots \mathrm{d}x_{(d)} \\ =& (2\pi)^{d/2} \rho \xi^d \ . \end{aligned} \tag{22}$$

- *Rectangular kernel.* This kernel is the basis for Random Geometric Graphs [10]. It reads

$$f\left(\frac{|x_i - x_j|}{\xi}\right) = \begin{cases} 1, & |x_i - x_j| \leq \xi, \\ 0, & |x_i - x_j| > \xi, \end{cases} \tag{23}$$

with ξ again being the dispersal distance. Integrating over the whole Euclidean space, we are actually obtaining the volume of a d-dimensional sphere of radius ξ. The effective number of neighbors is therefore given by

$$n_e = \frac{\pi^{d/2}}{\Gamma\left(d/2 + 1\right)} \rho \xi^d. \tag{24}$$

It is surprising to notice that, for $d = 1$ and $d = 2$, the expected n_e is equal for two different kernels. Specifically, when $d = 1$ the exponential and the rectangular kernel have the same value of n_e, while in two dimensions the Gaussian and exponential kernel have the same value of n_e. This means that two large matrices built with different kernels can have approximately the same λ.

Since the effective number of neighbors n_e is an approximation for the average row sum of M, and this quantity is itself a lower bound for the metapopulation capacity λ, we used simulations to see how well the approximation works in practice. Fig. S2 shows λ against n_e for various dispersal kernels and number of dimensions d. The region Ω is taken to be a d-dimensional cube of unit volume, and the dispersal distance ξ is varied between 0 and 0.2. The effective number of neighbors n_e is obtained by evaluating the

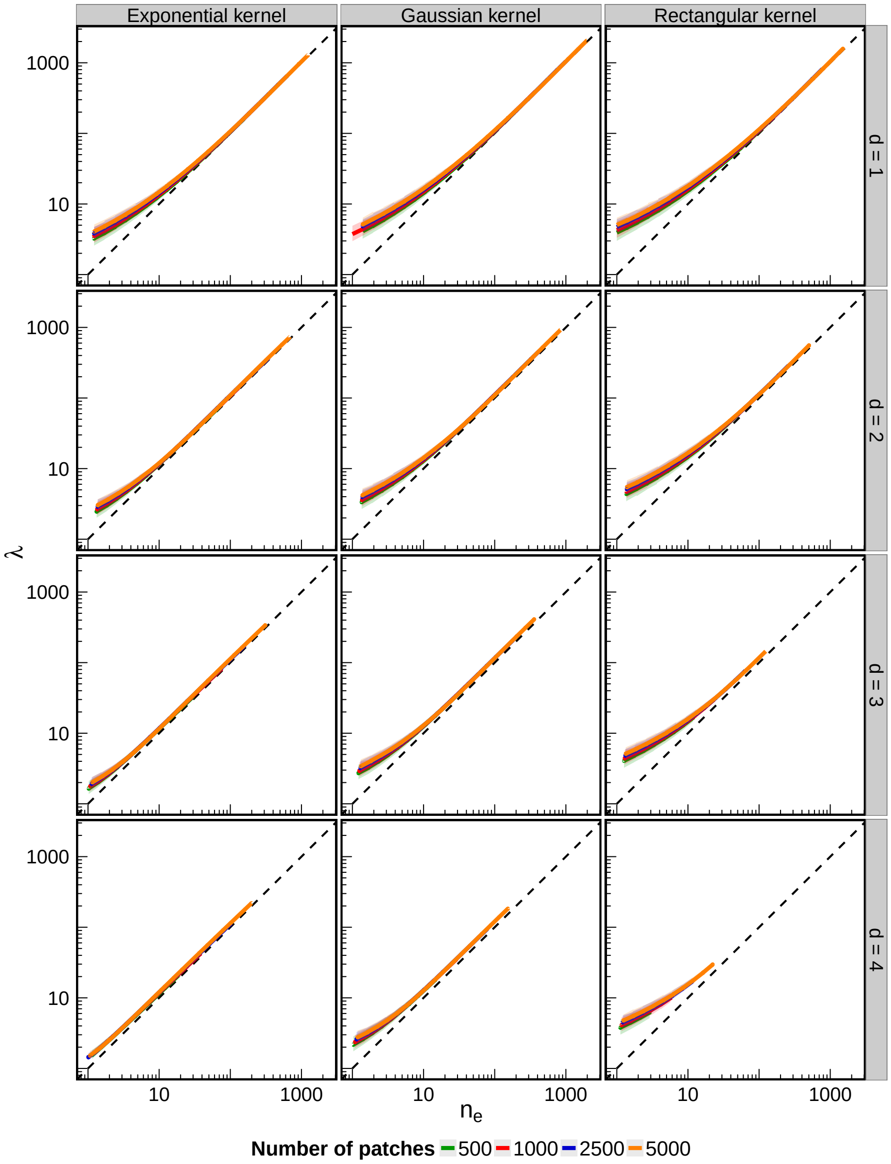

Fig. S2 Approximating the metapopulation capacity λ using the effective number of neighbors n_e. There are 4×3 insets; the rows correspond to the dimension d, and the columns to different dispersal kernels. Each curve is obtained from a set of simulations with given d, kernel, number of patches (colors), and dispersal distance ξ (varied between 0 and 0.2). For each parameter combination, we performed 1000 simulations. In each simulation patches of identical value are scattered uniformly in the $d-$dimensional unit cube, M is calculated using the parameters, λ is obtained by numerically finding the leading eigenvalue of M, and n_e is obtained by performing the integral in Eq. (17). The approximation is conservative when n_e is small, and is very accurate for larger values.

integral Eq. (17) numerically within the unit cube, while λ is numerically calculated as the actual leading eigenvalue of the Euclidean Random Matrix M.

Fig. S2 shows that, unless the effective number of neighbors is very low, the approximation is very good. Even when n_e is small, the approximation errs on the conservative side: the actual leading eigenvalue is in fact larger than predicted, leading to a greater likelihood of persistence than n_e predicts.

To better show the difference between the two quantities, Fig. S3 shows the value $(\lambda - n_e)/n_e$ against n_e, i.e., the relative deviation of λ from n_e. Interestingly, this relative deviation seems to satisfy a simple power-law relationship, with an exponent of approximately $-1/4$. Despite the fact that we do not have an analytic explanation for this empirical result, Fig. S3 shows that this seems to be a very robust pattern, with potential implications for further developments in the theory of Euclidean Random Matrices.

6 The effect of randomly arranging patches

The results above are found when patches are uniformly distributed over the landscape. However, often the case of regular arrangement of patches is considered in the literature [16-18]. Here we investigate the effects of a regular arrangement of the patches in a perfect grid, and how persistence is influenced by random arrangements.

Starting from patches perfectly arranged in a $d-$dimensional grid, we choose a nonnegative number η and perturb the coordinates of every patch around their original values by a random number drawn from the range $\pm\eta$ with a uniform distribution. Small values of η lead to a patch arrangements that are almost regular, while a large enough η means that the patch distribution is essentially random, as in the case studied above (Section 4). We continuously increase η from zero up, and for each of its values calculate λ. Since the perturbation is random, we perform 1000 replicate simulations for every value of η.

In these simulations we set $d = 2$ and Ω to be a square of unit area. Since coordinates may become less than zero or greater than one when perturbing the grid, we took the modulus of each coordinate with respect to one to contain all patches within the unit square (reflective perturbations). We chose a Gaussian dispersal kernel and varied the dispersal distance ξ between 0 and 0.2. The total number of patches N was set to 2500, i.e., the unperturbed case consisted of a regular 50×50 grid of patches.

Fig. S4 shows the results. In each and every case, the metapopulation capacity λ increases with η, regardless of the value of the dispersal distance. The dependence of λ on η exhibits a saturating trend: the increase is rapid at first, but then λ settles down at a constant for larger perturbations. This means that even slight deviations from a perfect grid make the system behave as if the patches were randomly scattered. We can also see that the more regular the grid, the better approximation n_e provides for λ.

6.1 Analytical argument

In the case of monotonically decreasing dispersal kernels one can justify the above results analytically. As shown in Section 5, the metapopulation capacity for a random arrangement of patches is bounded from below by the average row sum of the matrix. In the case of randomly distributed points we can approximate the row sum by evaluating the integral of Eq. (17). In the case of a regular arrangement of points the metapopulation capacity is exactly equal to the average row sum of the matrix. Therefore, if we indicate the (expected) metapopulation capacity in the random case by λ_{R} and the expected row sum of the matrix by $\mathbb{E}(r_R)$, and we assume a large landscape ($\xi \ll L$, with L effectively infinite), we have

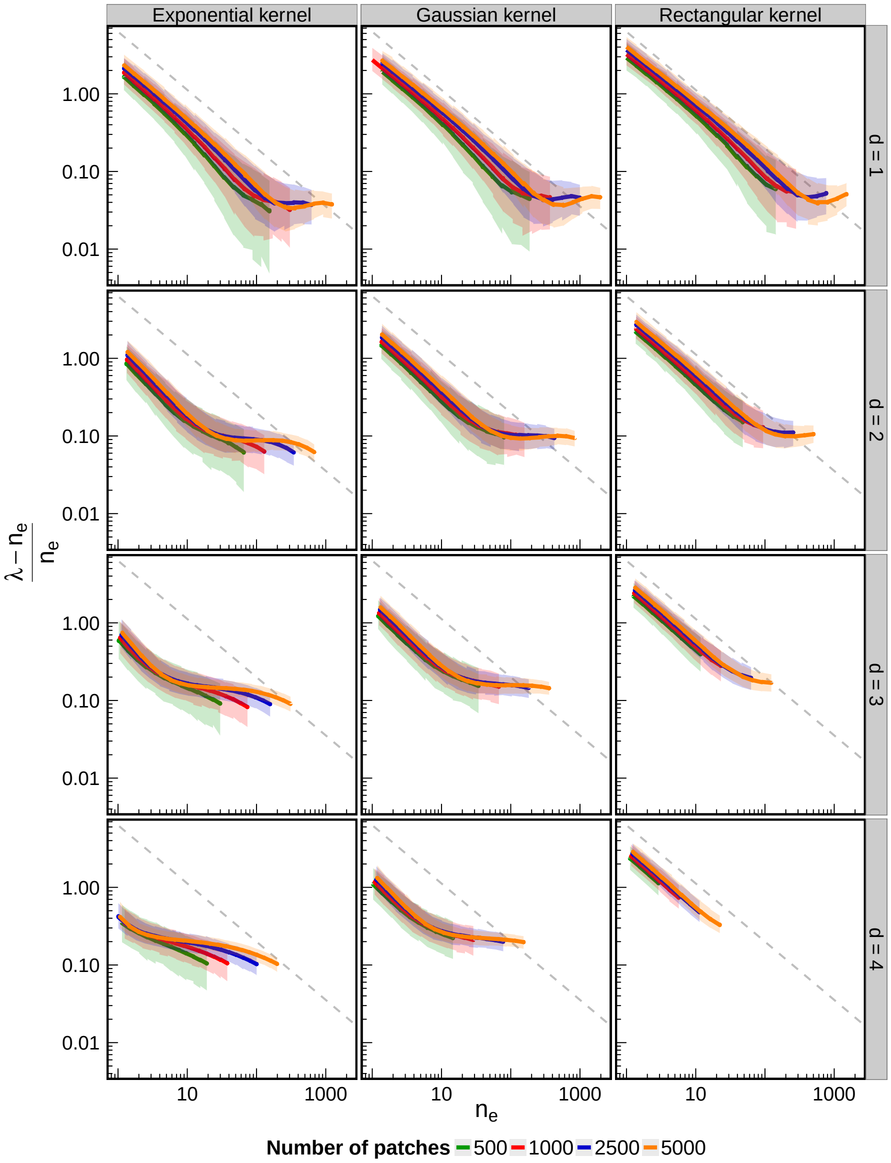

Fig. S3 Relative deviation of the metapopulation capacity λ from the effective number of neighbors n_e. Zero deviation means the effective number of neighbors provides a perfect estimate of the metapopulation capacity. Methods and notation as in Fig. S2. The gray dashed lines indicate a power-law relationship with exponent $-1/4$. The actual results approximate this ideal curve very well, suggesting the simple rule $(\lambda - n_e)/n_e \approx Q(d, f)n_e^{-1/4}$. The factor Q depends on the number of dimensions d and the general form of the dispersal kernel, but not on the dispersal distance ξ or the number of patches N. Rearranging, we get the improved approximation $\lambda \approx n_e + Q(d, f)n_e^{3/4}$ for the metapopulation capacity in place of $\lambda \approx n_e$. Efficient practical usage of this improved formula would of course require a knowledge of the function Q.

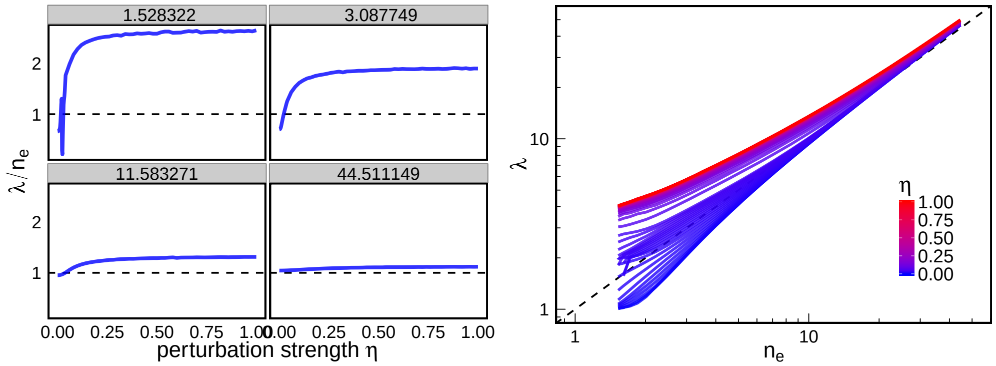

Fig. S4 The effect of a grid structure versus randomly distributed patches on the metapopulation capacity λ and the approximation $\lambda \approx n_e$. The perturbation size η measures the deviation of the patch distribution from a regular layout: $\eta = 0$ corresponds to a perfect grid, $\eta = 1$ corresponds to randomly distributed patches. Left panel: λ as a function of increasing η; this saturating pattern was recovered in every simulation. The λ on the vertical axis is rescaled using the expected effective number of neighbors n_e. Small deviations from the perfect grid distribution cause an increase in the metapopulation capacity, after which the curve quickly saturates. In other words, even mild deviations from a perfect grid behave as if the patches were randomly scattered. Right panel: approximation of λ using n_e as a function of the perturbation size. The dashed line is the identity line $n_e = \lambda$. One can see that the larger the perturbation, the more conservative the estimate, regardless of the magnitude of n_e. For large values of n_e, the separate curves for various perturbation sizes converge so that $n_e \approx \lambda$.

$$\mathbb{E}(r_R) = 2\int_0^\infty f(x)\,\mathrm{d}x \leq \lambda_\mathrm{R}\ , \tag{25}$$

where for simplicity we assumed $d = 1$. In the case of a grid arrangement the metapopulation capacity λ_G is equal to the expected row sum $\mathbb{E}(r_G)$

$$\mathbb{E}(r_G) = 2\sum_{i=1}^{\infty} f(i) = \lambda_\mathrm{G} \tag{26}$$

for the regular one-dimensional grid. These relations are true without any assumptions on the form of the dispersal kernel. In Section 9 we consider the general case of non-monotonically decreasing dispersal kernels, while in the following we focus on monotonically decreasing ones.

One can prove [42] that if $f(x)$ is monotonically decreasing,

$$\int_1^\infty f(x)\,\mathrm{d}x \leq \sum_{i=1}^{\infty} f(i) \leq f(1) + \int_1^\infty f(x)\,\mathrm{d}x \tag{27}$$

and

$$f(1) \leq \int_0^1 f(x)\,\mathrm{d}x. \tag{28}$$

By combining these inequalities with the definitions of λ_G and λ_R, we obtain

$$\lambda_{\mathrm{G}} = 2\sum_{i=1}^{\infty} f(i) \leq 2\int_{0}^{\infty} f(x)\,\mathrm{d}x \leq \lambda_{\mathrm{R}}, \tag{29}$$

showing that for monotonically decreasing dispersal kernels a random arrangement of patches always leads to a larger metapopulation capacity than a grid arrangement.

7 Localization

In this section we discuss two further features of metapopulation structure over random fragmented landscapes. The first is variance in patch importance, meaning that some habitat patches are more important for the metapopulation's persistence than others. The second is spatial localization: patches with high occupancy probabilities p_i tend to be close together in space.

The components of the leading eigenvector of M provide a measure of the importance of a patch for persistence. It can be shown [27] that the relative change in the metapopulation persistence due to the removal of patch i is approximately equal to w_i^2, where w_i is the ith component of the leading eigenvector. As we have shown above, the eigenvector is also related, in the limit $\delta \approx \lambda$, to the stationary patch occupancy probabilities p_i. High variance in the eigenvector components therefore corresponds to heterogeneity in patch importance.

To study the variance in patch importance quantitatively, we use a metric applicable to any nonzero vector w with nonnegative components that is normalized to have length 1,

$$\sum_{i=1}^{N} w_i^2 = 1, \tag{30}$$

where w_i is the ith component of the vector w. The *inverse participation ratio* (IPR [43]) is defined as

$$\mathrm{IPR} = \sum_{i=1}^{N} w_i^4 \ , \tag{31}$$

and measures the heterogeneity of eigenvector components. In fact IPR is related to the variance of w^2, being $\mathrm{Var}(w^2) = (1/N)\sum_{i=1}^{N} w_i^4 - (1/N)^2$. Here we rescale this metric so that its values fall between 0 and 1:

$$\Psi = \frac{N \times \mathrm{IPR} - 1}{N-1} = \frac{N\sum_{i=1}^{N} w_i^4 - 1}{N-1}. \tag{32}$$

When patches are arranged in a perfect grid, we do not expect any variance in the w_i (with the exception of the possible small influence of boundary effects). To measure how patch occupancies changes for disordered systems, we first perturbed a regular grid of patches in exactly the same way as described in Section 6. In Fig. S5 we show the scaled inverse participation ratio Ψ of the normalized leading eigenvector w against the metapopulation capacity λ for various values of the perturbation size η. We see that increasing randomness leads to higher variation in all cases.

This simulation sheds light on the cause for the discrepancy between λ and n_e observed in Fig. S2 and S3. We have shown that n_e approximates λ very well, except when n_e is very small. The relationship between the two quantities is of course not expected to be perfect since the effective number of neighbors only provides a lower bound for λ, as discussed in Section 5. The only case when the lower bound actually

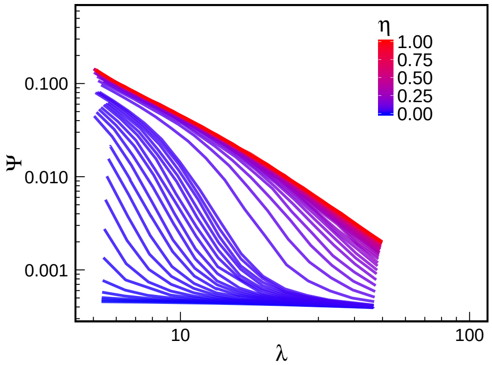

Fig. S5 The effect of a grid structure versus randomly distributed patches on the variance in patch occupancies. The scaled inverse participation ratio Ψ of the normalized leading eigenvector w, defined by Eq. (32), is plotted against the metapopulation capacity λ. Notation and simulation setup are as in Fig. S4, explained in detain in Section 6. The perturbation size η measures the deviation of the patch distribution from a regular layout: $\eta = 0$ corresponds to a perfect grid, $\eta = 1$ corresponds to randomly distributed patches. Ψ increases with the perturbation size η (increasing randomness increases Ψ), and decreases with the metapopulation capacity. Note that, since our formula for Ψ depends on the approximation Eq. (11), i.e., the $\lambda \approx \delta$ limit, the metapopulation is still close to extinction even for large values of the metapopulation capacity.

holds with strict equality is when w is completely uniform, i.e., is proportional to the vector $(1, 1, \ldots)$. But this implies that $\Psi = 0$, corresponding to the lack of any variation in patch occupancies. Any deviation from this situation leads to more variance and therefore a worse approximation due to Eq. (14). Therefore, any discrepancy between λ and n_e is strictly due to this variance.

Instead of relating Ψ to the degree of randomness in the distribution of patches across the landscape, one may also ask how it relates to the metapopulation capacity itself. To answer this question, we generated 1000 matrices M for each combination of the following parameters: N (which could take on the values 500, 1000, 2500, and 5000), d (between 1 and 4), kernel (Exponential, Gaussian, and Rectangular), and dispersal distance ξ (taking on 84 possible values between 0 and 0.2). In each case we determined Ψ and λ for each M. The scaled inverse participation ratio Ψ was then calculated. Fig. S6 shows the results. We can see that Ψ is inversely related to λ in all cases: a larger metapopulation capacity leads to less variance in patch occupancies. Though this result might seem self-evident, we have assumed $\lambda \approx \delta$, and therefore high values of the metapopulation capacity do not in any way imply strong metapopulation persistence—on the contrary, regardless of the magnitude of λ, the metapopulation is very close to the extinction threshold.

We also performed a complementary analysis, where we looked at the average patch occupancy probability $\overline{p}$ of the metapopulation, where the bar denotes the expected value. Since in the $\lambda \approx \delta$ limit Eq. (11) describes the equilibrium occupancy probabilities p_i, the average occupancy is given by

$$\overline{p} = \frac{1}{N} \sum_{i=1}^{N} p_i = \frac{\lambda - \delta}{\lambda} \left(\sum_{j=1}^{N} w_j^3 \right)^{-1} \frac{1}{N} \sum_{i=1}^{N} w_i. \quad (33)$$

Denoting terms related to the leading eigenvector with F:

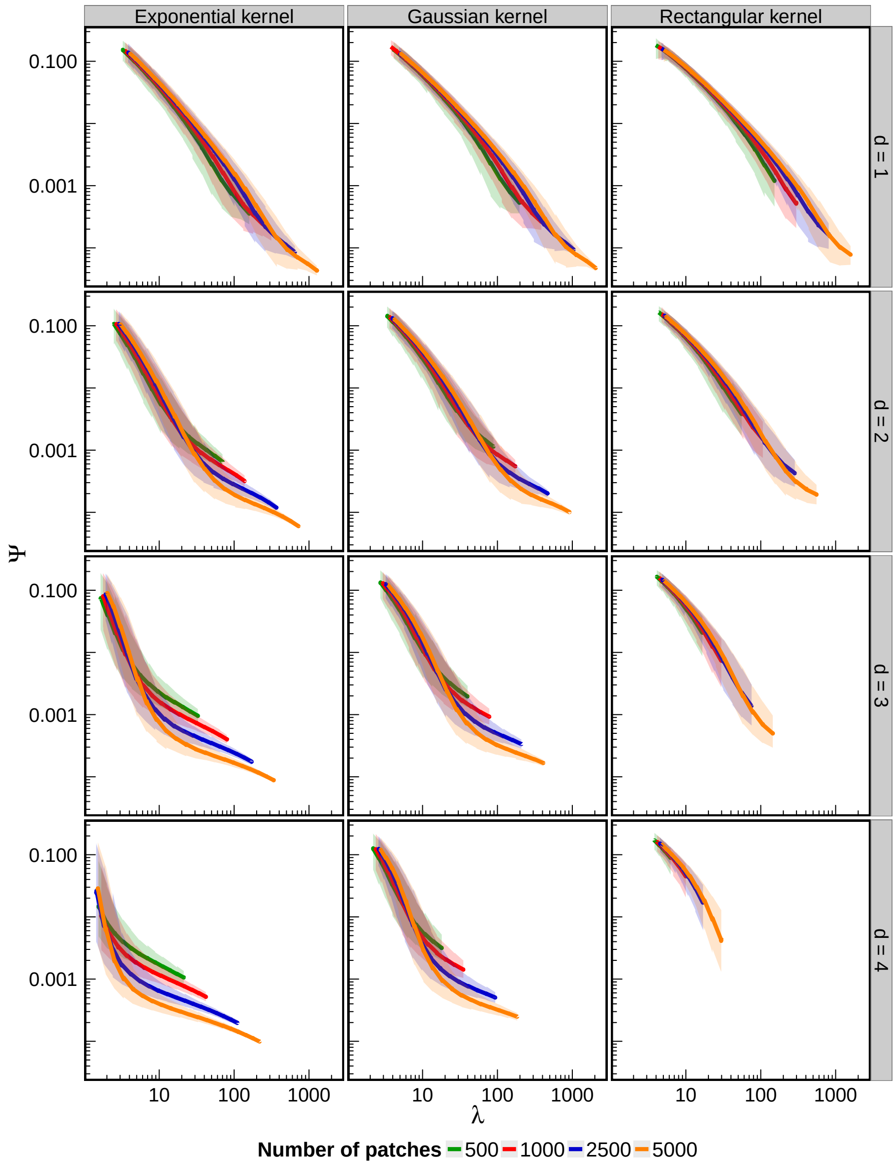

Fig. S6 The scaled inverse participation ratio Ψ as a function of the metapopulation capacity in the $\lambda \approx \delta$ limit. Methods and notation as in Fig. S2. Ψ diminishes with an increasing metapopulation capacity in all cases.

$$\overline{p} = \frac{\lambda - \delta}{\lambda} F, \tag{34}$$

where

$$F = \left(\sum_{j=1}^{N} w_j^3 \right)^{-1} \frac{1}{N} \sum_{i=1}^{N} w_i. \tag{35}$$

This quantity depends only on the leading eigenvector of M. It is similar to Ψ, but while Ψ is a generic measure of the variance, F is more directly relevant to the $\lambda \approx \delta$ case in our model, because of the approximation Eq. (11). The minimum value F is achieved for $F_{\min} = 1/N$, which happens when we have maximal variance, i.e., one single component of the leading eigenvector w is equal to 1 and all the others are zero. In contrast, the maximum possible value of F is one, taken on in the absence of any variation, i.e., when $w_i = 1/\sqrt{N}$ for all i:

$$F_{\max} = \frac{\sqrt{N^3}}{N} \frac{1}{N} \frac{N}{\sqrt{N}} = 1. \tag{36}$$

Using the same methodology as in the case of Ψ, we can plot F against the metapopulation capacity λ. The results are seen in Fig. S7. In every case shown, F increases with λ, and the curves saturate at $F = 1$, where there is no longer any variation in the patch occupancies.

Fig. S6 and Fig. S7 together imply that the variance in patch importance is diminished by having a very large metapopulation capacity, irrespective of the fact that, since δ is close to λ, the patch occupancy probabilities are of course not going to be very large. It is also seen however that this variance only tends to disappear for very large values of the metapopulation capacity. This implies that, unless metapopulations are very strongly persistent, variance in patch importance is a natural consequence of metapopulation dynamics with randomly distributed patches.

Having established that there is variation in the importance of patches for metapopulation persistence, we can also ask whether patches with similar importance tend to form spatial clusters, i.e. whether patches of similar importance are close together on the landscape. To answer this question, we measured the correlation between closeness and importance using Moran's index [44]. Independent of n_e, this index turns out to be positive and significant (p-value always less than 10^{-6}), i.e. close patches have similar importance. This positive correlation is expected, since in terms of network theory, the importance of a node is measured by the eigenvector's components (the so-called eigenvector centrality). It directly follows from the definition of eigenvector centrality (important nodes are nodes connected with important nodes) that strongly connected patches have similar importance. But the patches that are going to be strongly connected are the ones close in space, since the strength of connection depends only on the distance between patches in our Euclidean Random Matrix framework. This argument already implies a positive Moran's index—which we indeed do find.

8 The effect of patch heterogeneity

So far we have assumed that all patches have the same value, i.e., in Eq. (4) the A_i are all equal. In fact, we have assumed $A_i = 1$; however, for any constant patch value $A_i = a$ one can rescale the equilibrium equation Eq. (9) by dividing both sides by a^2 and treating δ/a^2 as a new effective δ. Here we introduce variable patch values.

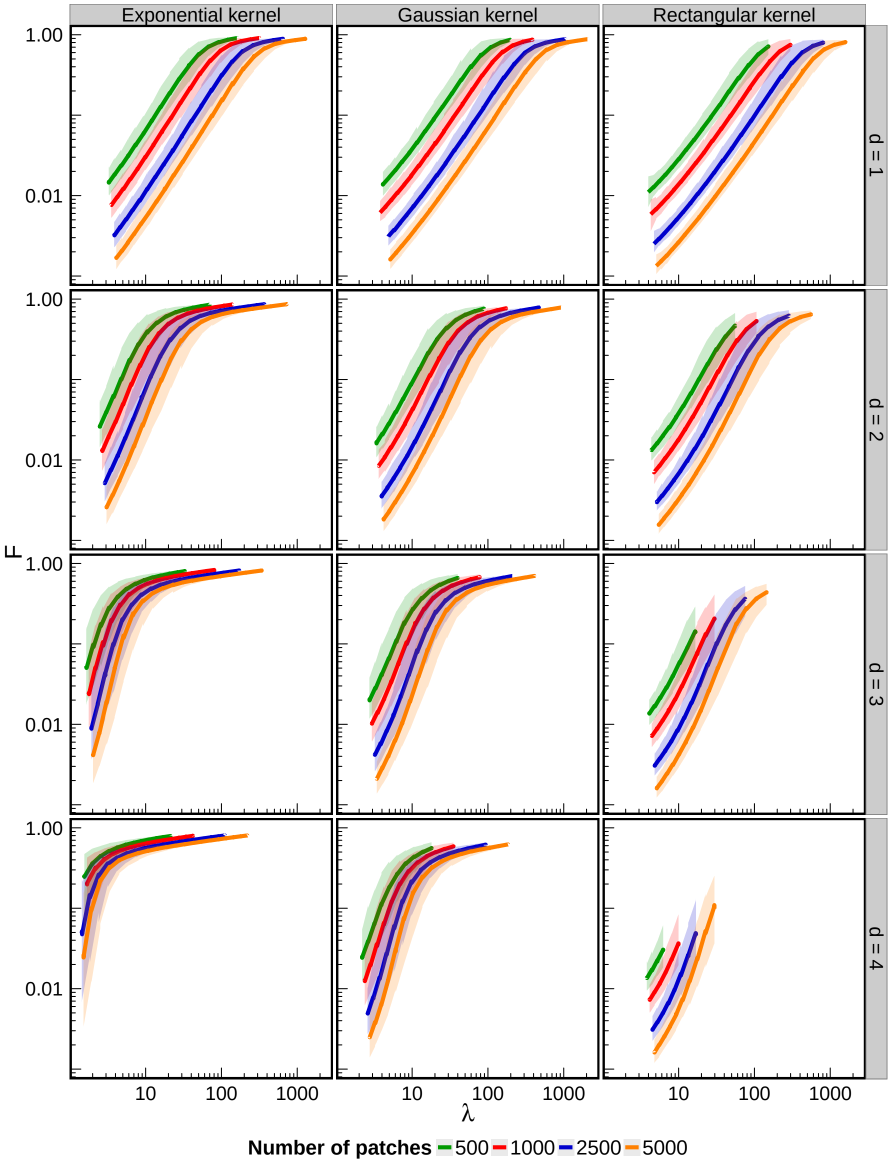

Fig. S7 A different measure of the variance in patch importance as a function of the metapopulation capacity, in the $\lambda \approx \delta$ limit. Here we use the quantity F, defined in Eq. (35), on the vertical axis. This measure of the variance in patch importance is more directly taylored to our particular model, as F is used in the approximation Eq. (11). Otherwise, methods and notation are as in Fig. S6. Again, the conclusion that the variance in patch importance always increases with the metapopulation capacity λ holds.

To explore their effects on metapopulation structure and persistence, we randomized the A_i by drawing them independently from a scaled beta distribution with mean 1 and variance σ^2 (again, without loss of generality).

We can use Eq. (7) with $q = 2$ to derive an improved approximation for the metapopulation capacity when the patch values are variable:

$$\lambda \geq \frac{\sum_{i=1}^{N}\sum_{j=1}^{N}\left(M^2\right)_{ij}}{\sum_{i=1}^{N}\sum_{j=1}^{N}M_{ij}}, \tag{37}$$

or, writing out the matrix multiplication in the numerator,

$$\lambda \geq \frac{\sum_{i=1}^{N}\sum_{j=1}^{N}\sum_{k=1}^{N}M_{ik}M_{kj}}{\sum_{i=1}^{N}\sum_{j=1}^{N}M_{ij}}. \tag{38}$$

Substituting in Eq. (4) and using the notation $f_{ij} = f\left(|x_i - x_j|/\xi\right)$ when $i \neq j$ and 0 for $i = j$,

$$\lambda \geq \frac{\sum_{i=1}^{N}\sum_{j=1}^{N}\sum_{k=1}^{N}A_iA_jA_k^2 f_{ik}f_{kj}}{\sum_{i=1}^{N}\sum_{j=1}^{N}A_iA_jf_{ij}}, \tag{39}$$

where the matrix f is a Euclidean Random Matrix.

The matrix f has zeros on the diagonal, and therefore the sum in the numerator always yields zero whenever $k = i$ or $k = j$. Since f is a function of independently drawn random points in space, and the patch values are also independently drawn, the numerator can be treated as an averaging over the patches via the index k. Let us define the quantity

$$L_k = \sum_{i=1}^{N}\sum_{j=1}^{N}A_iA_jf_{ik}f_{kj}. \tag{40}$$

We then have

$$\lambda \geq \frac{\sum_{k=1}^{N}A_k^2L_k}{\sum_{i=1}^{N}\sum_{j=1}^{N}A_iA_jf_{ij}}, \tag{41}$$

or

$$\lambda \geq \frac{N\overline{A^2L}}{\sum_{i=1}^{N}\sum_{j=1}^{N}A_iA_jf_{ij}}, \tag{42}$$

where the bar denotes averaging over the patches. Due to independence, we have $\overline{A^2L} = \overline{A^2}\,\overline{L}$. The quantity $\overline{L}$ can be written

$$\begin{aligned}\overline{L} =& \frac{1}{N}\sum_{k=1}^{N}\sum_{i=1}^{N}\sum_{j=1}^{N}A_iA_jf_{ik}f_{kj} = \frac{1}{N}\sum_{k=1}^{N}\left(\sum_{i=1}^{N}A_if_{ik}\right)\left(\sum_{j=1}^{N}A_jf_{kj}\right)\\ =& \frac{1}{N}\sum_{k=1}^{N}\left(\overline{A}\sum_{i=1}^{N}f_{ik}\right)\left(\overline{A}\sum_{j=1}^{N}f_{kj}\right) = \frac{(\overline{A})^2}{N}\sum_{i=1}^{N}\sum_{j=1}^{N}\left(f^2\right)_{ij},\end{aligned} \tag{43}$$

where we used the independence of A and f again. The denominator of Eq. (42) can be written

$$\sum_{i=1}^{N}\sum_{j=1}^{N} A_i A_j f_{ij} = \sum_{i=1}^{N} A_i \sum_{j=1}^{N} A_j f_{ij} = \overline{A} \sum_{i=1}^{N}\sum_{j=1}^{N} A_j f_{ij} = (\overline{A})^2 \sum_{i=1}^{N}\sum_{j=1}^{N} f_{ij}. \tag{44}$$

Substituting Eqs. (43) and (44), we obtain

$$\lambda \geq \overline{A^2}\, \frac{\sum_{i=1}^{N}\sum_{j=1}^{N} \left(f^2\right)_{ij}}{\sum_{i=1}^{N}\sum_{j=1}^{N} f_{ij}}. \tag{45}$$

We discussed in Section 2 that, while both Eq. (6) and Eq. (7) (with $q = 2$) approximate λ, the latter is always greater than or equal to the former:

$$\frac{\sum_{i=1}^{N}\sum_{j=1}^{N} \left(f^2\right)_{ij}}{\sum_{i=1}^{N}\sum_{j=1}^{N} f_{ij}} \geq \frac{\sum_{i=1}^{N}\sum_{j=1}^{N} f_{ij}}{N}. \tag{46}$$

Therefore we may substitute this into Eq. (45) and the inequality will still hold with

$$\lambda \geq \frac{\overline{A^2}}{N} \sum_{i=1}^{N}\sum_{j=1}^{N} f_{ij}. \tag{47}$$

But the above sum divided by N is simply the effective number of neighbors n_e, as discussed in Section 5. We therefore have

$$\lambda \geq \overline{A^2}\, n_e. \tag{48}$$

Since we are drawing the patch values independently from a distribution with mean 1 and variance σ^2, and $\overline{A^2} = (\overline{A})^2 + \mathrm{Var}(A)$, we have $\overline{A^2} = 1 + \sigma^2$. We therefore obtain

$$\lambda \geq (1 + \sigma^2) n_e. \tag{49}$$

We tested how the original $\lambda \geq n_e$ and the new $\lambda \geq (1 + \sigma^2)n_e$ approximations perform as a function of the patch variability σ. The left panel of Fig. S8 shows the former, the right panel the latter approximation. On the left panel, for small values of σ we see exactly the same situation found in Fig. S2, but for large values of σ the effective number of neighbors starts to severely underestimate the metapopulation capacity. On the right panel however, we see that as long as $(1 + \sigma^2)n_e$ is not very small, this quantity approximates the metapopulation capacity very well even for large patch variability.

As done in Fig. S6, we measure the variance in patch importance as a function of the metapopulation capacity (Fig. S9). Just as before, lower values of λ lead to a higher variance. More importantly, patch variability clearly exacerbates this effect: for any value of the metapopulation capacity, adding variation to the value of the patches generates more variance in patch importance than expected for uniform constant patch values.

Since for constant patch values $\sigma = 0$, the approximation $\lambda \geq (1 + \sigma^2)n_e$ is an extension to our previous result $\lambda \geq n_e$, encompassing it as a special case.

The metapopulation capacity does not depend on the full distriution of patch values, but only on its mean and variance. Here we show why we can neglect moments higher than two. Eq. (7), in the case of $q = 3$, reads

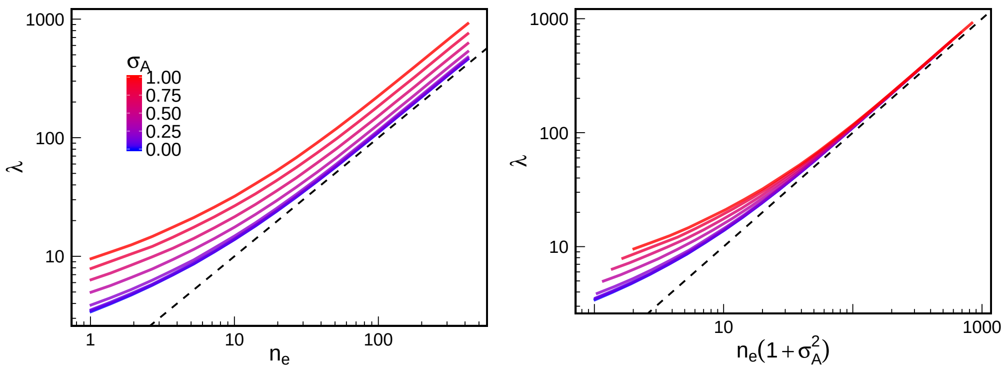

Fig. S8 The effect of nonconstant patch values on the approximation $\lambda \approx n_e$. On the left panel we plot the metapopulation capacity (obtained by computing λ numerically) against the effective number of neighbors n_e, calculated via the integral in Eq. (17). The patch values were drawn from a rescaled beta distribution with mean 1 and standard deviation σ. The matrices M were generated with $N = 2500$, $d = 2$, a Gaussian dispersal kernel, the dispersal distance ξ varying between 0 and 0.2, and σ varying between 0 and 1. For every parameter combination, 1000 simulations were performed. As seen on the plot, the greater the variability in patch value, the more n_e underestimates λ. The right panel shows the exact same information, except the metapopulation capacity is plotted against $n_e(1 + \sigma^2)$ instead of just n_e (Section 8). The approximation is significantly improved by including the term $(1 + \sigma^2)$.

$$\lambda \geq \frac{\sum_{i=1}^{N} \sum_{j=1}^{N} \sum_{k=1}^{N} \sum_{l=1}^{N} A_i f_{ij} A_j^2 f_{jk} A_k^2 f_{kl} A_l}{\sum_{i=1}^{N} \sum_{j=1}^{N} \sum_{k=1}^{N} A_i f_{ij} A_j^2 f_{jk} A_k}. \tag{50}$$

Using the fact that $f_{ii} = 0$, we can partition the sum in the numerator into different contributions, corresponding to 1) the indices i, j, k and l being all different; 2) $i = k$ or $j = l$; and 3) $i = l$. The leading contribution in N will be the one corresponding to all the indices being different and will be proportional to the square of the mean of A squared. If we consider only this leading contribution, then using the fact that the denominator is proportional to $\overline{A^2}$ (Eq. 43), we obtain again a linear dependence of λ on $\overline{A^2}$. The terms we neglected in this approximation will be proportional to the third or the fourth moments of the distribution of patch values. If those moments are finite it is safe to neglect them in the limit of large N. Therefore we expect our approximation to hold as long as the moments of the distribution of patch values are finite and to break down if the distribution of patch values have non-finite moments (e.g., power-law tails).

9 Effects of nonmonotonic dispersal kernels

In previous sections we considered only monotonically decreasing dispersal kernels. In that case, the closer two patches are, the higher the rate of dispersal will be between them. In this section we consider nonmonotonic dispersal kernels. In this case dispersal rate between two patches is maximal at some given finite distance.

We used the following function for modeling nonmonotonic dispersal kernels:

$$f\left(\frac{|x_i - x_j|}{\xi}\right) = \frac{\Gamma(\alpha+\beta)}{\Gamma(\alpha)\Gamma(\beta)} \left(\frac{|x_i - x_j|}{\xi}\right)^{\alpha-1} \left(1 - \frac{|x_i - x_j|}{\xi}\right)^{\beta-1} \tag{51}$$

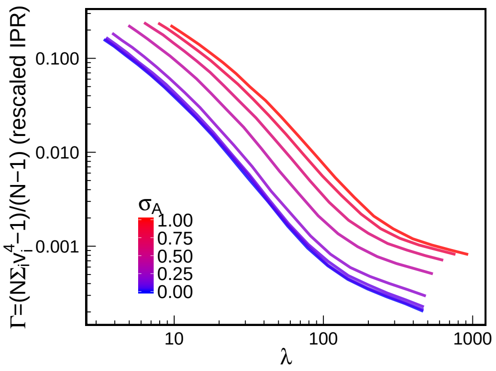

Fig. S9 The effect of nonconstant patch values on the variance in patch importance. We plot the scaled inverse participation ratio Ψ, defined in Eq. (32), against the metapopulation capacity λ. Parameter combinations were chosen and simulations were done exactly as in Fig. S8. Each curve corresponds to a different value of σ. The plot shows that Ψ always increases with increasing variability.

for $|x_i - x_j| \leq \xi$ and 0 for $|x_i - x_j| > \xi$, where Γ is the gamma function. The parameters α and β control the shape of the distribution and the location of the mode. When $|x_i - x_j| \leq \xi$, Eq. (51) reduces to a beta-distribution. The choice $\alpha = \beta = 1$ corresponds to a rectangular kernel.

It turns out that all our previous results are robust to changing the kernel to nonmonotonic ones. Fig. S10 shows the metapopulation capacity λ and the effective number of neighbors n_e for various nonmonotonic dispersal kernels. Our approximation perfoms similarly as in the case of strictly decreasing dispersal kernels (see fig. S2).

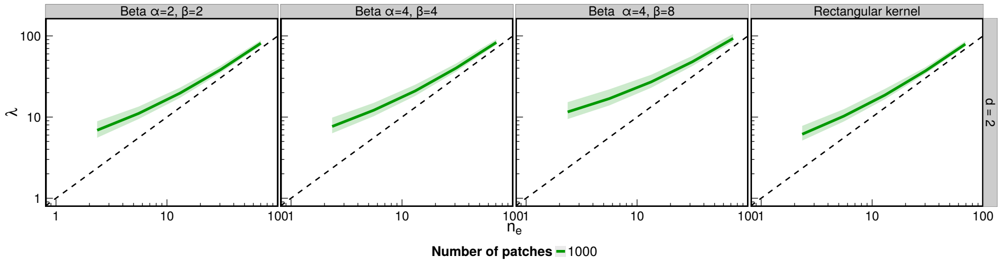

Fig. S10 Approximating the metapopulation capacity λ using the effective number of neighbors n_e for nonmonotonic dispersal kernels. Different insets correspond to different kernels as defined by Eq. (51). Otherwise, the figure has been obtained in the same way as Fig. S2, with $d = 2$, $N = 1000$, and $\sigma = 0$.

9.1 The effect of randomly arranging patches

In Section 6 we showed that, in the case of monotonically decreasing dispersal kernels, the metapopulation capacity of a random arrangement of patches is always larger than for a grid arrangement. In this section we consider the case of nonmonotonically decreasing dispersal kernels, where the results of Section 6 no longer hold. We consider the parameterization of Eq. (51) with $\alpha = \beta$, having kernels depend only on one parameter α. By increasing the value of α we obtain dispersal kernels that are more and more peaked around the nearest neighbor-patches on the grid.

In the $d = 1$ case, the lower bound for the metapopulation capacity in a random arrangement of patches can be obtained by integrating Eq. (51). When periodic boundary conditions are considered, one obtains $\lambda_{\mathrm{R}} \geq 2$ (see Section 6). In the grid case the sum shown in Section 6 is equal to

$$\lambda_{\mathrm{G}} = \frac{2^{3-2\alpha}}{B(\alpha,\alpha)}. \tag{52}$$

Comparing the lower bound for the random arrangement and the metapopulation capacity of a grid, we obtain that $\lambda_{\mathrm{G}} \leq 2$ if $\alpha < \alpha_{\mathrm{c}} \approx 3.382\ldots$ In this case the random arrangement is guaranteed to have a larger metapopulation capacity. Note that if $\alpha > \alpha_{\mathrm{c}}$ we cannot say anything about which arrangement has a larger metapopulation capacity, as 2 is a lower bound for λ_{R}.

Fig. S11 shows the effect of regular versus random distribution of patches (see Section 6) for nonmonotonic dispersal kernels. Interestingly, the actual value of λ_{R} is always larger than λ_{G}.

The take-home message of this result is that, even in the case of non-monotonically decreasing dispersal kernels, given a region of space with a given number of patches, the metapopulation capacity λ is always larger for randomly distributed patches than for a regular grid. This has important consequences for conservation and landscape design: regularly spaced habitats turn out to be less efficient than randomly distributed ones. Random distributions lead to an increase in λ and therefore an increase in the overall persistence likelihood of the metapopulation as a whole.

10 Approximating p_i

10.1 Approximation 1: strongly persistent metapopulations

We want to approximate p_i assuming $\lambda \gg \delta$, where λ is the metapopulation capacity, i.e., the leading eigenvalue of M.

When δ is sufficiently small, we may look for the solution to Eq. (9) in the form of a power series in δ:

$$p_i = p_i^{(0)} + \delta p_i^{(1)} + \delta^2 p_i^{(2)} + \ldots \tag{53}$$

Substituting this expression into Eq. (9) yields

$$\begin{aligned} &\left(1 - p_i^{(0)} - \delta p_i^{(1)} - \delta^2 p_i^{(2)} - \ldots\right) \sum_{j=1}^{N} M_{ij} \left(p_j^{(0)} + \delta p_j^{(1)} + \delta^2 p_j^{(2)} + \ldots\right) \\ &= \delta \left(p_i^{(0)} + \delta p_i^{(1)} + \delta^2 p_i^{(2)} + \ldots\right). \end{aligned} \tag{54}$$

We can proceed by increasing powers of δ. Collecting terms of 0th order:

$$\left(1 - p_i^{(0)}\right) \sum_{j=1}^{N} M_{ij} p_j^{(0)} = 0, \tag{55}$$

which is satisfied either by $p_i^{(0)} = 0$ for all i (the solution describing an extinct population), or by $p_i^{(0)} = 1$ for all i. Moving on to terms of first order, we get, from Eq. (54),

$$\delta \left(1 - p_i^{(0)}\right) \sum_{j=1}^{N} M_{ij} p_j^{(1)} - \delta p_i^{(1)} \sum_{j=1}^{N} M_{ij} p_j^{(0)} = \delta p_i^{(0)}. \tag{56}$$

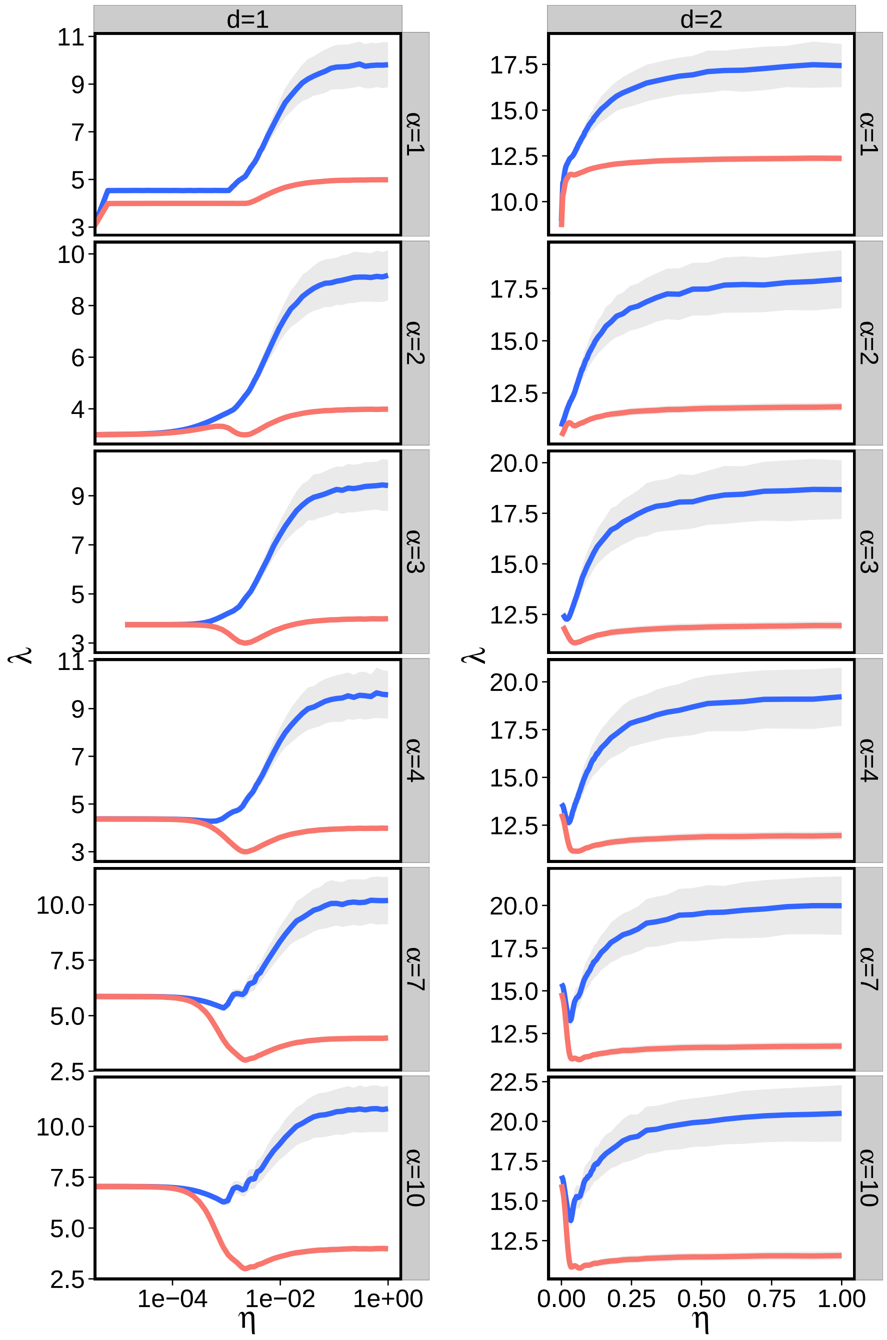

Fig. S11 The effect of a grid structure versus randomly distributed patches on the metapopulation capacity λ. The red line represents the effective number of neighbors, while the blue line is the actual eigenvalue. The parameter η quantifies the randomness of the patch arrangement, with $\eta = 0$ corresponding to the grid case and $\eta = 1$ to the random arrangement. Despite the fact that n_e decreases for some values of α as η increases, the metapopulation capacity λ_{R} is always larger than λ_{G}.

Using $p_i^{(0)} = 1$, this simplifies to

$$-\delta p_i^{(1)} \sum_{j=1}^{N} M_{ij} = \delta. \tag{57}$$

The first-order approximation is therefore given by

$$p_i^{(1)} = -\frac{1}{\sum_{j=1}^{N} M_{ij}}, \tag{58}$$

so p_i can be expressed from Eq. (53), to a first order approximation, as

$$p_i = 1 - \left(\frac{\delta}{\sum_{l=1}^{N} M_{il}} \right). \tag{59}$$

Now collecting terms of second order in Eq. (54):

$$-\delta^2 p_i^{(1)} \sum_{j=1}^{N} M_{ij} p_j^{(1)} - \delta^2 p_i^{(2)} \sum_{j=1}^{N} M_{ij} p_j^{(0)} = \delta^2 p_i^{(1)}. \tag{60}$$

Substituting in the values for $p_i^{(0)}$ and $p_i^{(1)}$ and simplifying by δ^2, we get

$$\frac{1}{\sum_{l=1}^{N} M_{il}} \sum_{j=1}^{N} M_{ij} \frac{1}{\sum_{k=1}^{N} M_{jk}} - p_i^{(2)} \sum_{l=1}^{N} M_{il} = -\frac{1}{\sum_{l=1}^{N} M_{il}}, \tag{61}$$

or

$$p_i^{(2)} = \frac{1}{\left(\sum_{l=1}^{N} M_{il} \right)^2} \left(1 + \sum_{j=1}^{N} M_{ij} \frac{1}{\sum_{k=1}^{N} M_{jk}} \right). \tag{62}$$

From Eq. (53), the equilibrium distribution p_i can therefore be written, to second order approximation, as

$$p_i = 1 - \left(\frac{\delta}{\sum_{l=1}^{N} M_{il}} \right) + \left(\frac{\delta}{\sum_{l=1}^{N} M_{il}} \right)^2 \left(1 + \sum_{j=1}^{N} M_{ij} \frac{1}{\sum_{k=1}^{N} M_{jk}} \right). \tag{63}$$

In practice, this approximation only works as long as the fraction $\delta / \sum_{l=1}^{N} M_{il}$ is small for all (or most) i. In that case however, the first-order approximation is already more than sufficient. Note that $\sum_{l=1}^{N} M_{il}$ is the row sum of row i in the matrix M_{ij}. As discussed in Section 2, this sum provides a lower bound for the leading eigenvalue, i.e., the metapopulation capacity λ. Therefore, this approximation works well whenever $\lambda \gg \delta$.

10.2 Approximation 2: metapopulations close to extinction

We now turn to our second approximation scheme, which assumes $\lambda \approx \delta$. Let λ_α be the αth eigenvalue, and $w_{\alpha,i}$ the ith component of the αth eigenvector of M:

$$\sum_{j=1}^{N} M_{ij} w_{\alpha,j} = \lambda_\alpha w_{\alpha,i}. \tag{64}$$

We may assume without loss of generality that the metapopulation capacity λ corresponds to λ_1. We write the stationary solution p_i as a linear combination of the eigenvectors:

$$p_i = \sum_{\alpha=1}^{N} k_\alpha w_{\alpha,i}, \tag{65}$$

where the k_α are appropriate coefficients, to be determined. Substituting into Eq. (9), we obtain

$$\sum_{\alpha=1}^{N} (\lambda_\alpha - \delta) k_\alpha w_{\alpha,i} - \sum_{\alpha=1}^{N} \sum_{\beta=1}^{N} k_\alpha k_\beta \lambda_\beta w_{\alpha,i} w_{\beta,i} = 0. \tag{66}$$

This equation is zero for all i, therefore multiplying it by $w_{\gamma,i}$ and summing over i yields the equivalent equation

$$(\lambda_\gamma - \delta) k_\gamma = \sum_{\alpha=1}^{N} \sum_{\beta=1}^{N} k_\alpha k_\beta \lambda_\beta \sum_{i=1}^{N} w_{\alpha,i} w_{\beta,i} w_{\gamma,i} \tag{67}$$

that holds for all γ. On the left hand side we have used the fact that the eigenvectors are orthogonal, since M is symmetric. On the right of hand side of Eq. (67) the summation over i can be written in the following equivalent way:

$$\sum_{i=1}^{N} w_{\alpha,i} w_{\beta,i} w_{\gamma,i} = I_{\alpha\beta} I_{\beta\gamma} \sum_{i=1}^{N} w_{\gamma,i}^3 + \epsilon_{\alpha\beta\gamma}, \tag{68}$$

where $I_{\alpha\beta}$ is the (α, β) entry of the identity matrix, and $\epsilon_{\alpha\beta\gamma}$ is a factor chosen so as to satisfy the equation above. If, in addition, we make the assumption that $\epsilon_{\alpha\beta\gamma}$ is small, it becomes possible to expand the solution with respect to this quantity:

$$k_\alpha = k_\alpha^{(0)} + (\text{corrections depending on } \epsilon_{\alpha\beta\gamma}) \,. \tag{69}$$

This will be possible when the eigenvectors have small overlap (i.e., the nonzero components are different for different eigenvectors). The $k_\alpha^{(0)}$ can be obtained by substituting Eq. (68) with $\epsilon_{\alpha\beta\gamma} = 0$ into Eq. (67):

$$(\lambda_\alpha - \delta)\, k_\alpha^{(0)} = \lambda_\alpha \left(k_\alpha^{(0)}\right)^2 \sum_{i=1}^{N} w_{\alpha,i}^3. \tag{70}$$

This equation has two solutions: $k_\alpha^{(0)} = 0$ and

$$k_\alpha^{(0)} = \frac{\lambda_\alpha - \delta}{\lambda_\alpha} \left(\sum_{i=1}^{N} w_{\alpha,i}^3 \right)^{-1}. \tag{71}$$

This approximation is obtained under the assumption that the eigenvectors do not overlap. Therefore it is consistent to assume that $k_\alpha^{(0)} = 0$ if $\lambda_\alpha < \delta$ and different from zero otherwise:

$$k_\alpha^{(0)} = \frac{\lambda_\alpha - \delta}{\lambda_\alpha} \left(\sum_{i=1}^{N} w_{\alpha,i}^3 \right)^{-1} \Theta(\lambda_\alpha - \delta), \tag{72}$$

where Θ is the Heaviside unit step function. By substituting this expression into Eq. (65), we get an approximation for the p_i when $\lambda \approx \delta$:

$$p_i = \sum_{\alpha=1}^{N} \left(\frac{\lambda_\alpha - \delta}{\lambda_\alpha} \left(\sum_{j=1}^{N} w_{\alpha,j}^3 \right)^{-1} \Theta(\lambda_\alpha - \delta) \right) w_{\alpha,i}. \tag{73}$$

If $\lambda \approx \delta$ with λ just barely exceeding δ, then, since λ is the leading eigenvalue, we do not expect any other eigenvalues to be greater than δ. Therefore, in this case, using the Heaviside function, Eq. (73) reduces to

$$p_i = \frac{\lambda - \delta}{\lambda} \left(\sum_{j=1}^{N} w_j^3 \right)^{-1} w_i, \tag{74}$$

where $w_i = w_{1,i}$ is the ith component of the leading eigenvector. This formula also appears in [20, Eq. 22].

References

40. Caswell H. Matrix population models: Construction, analysis and interpretation. 2nd edition. Sinauer Associates; 2001.

41. Otto SP, Day T. A Biologist's Guide to Mathematical Modeling in Ecology and Evolution. Princeton, USA: Princeton University Press; 2007.

42. Smarandache F. A Triple Inequality with Series and Improper Integrals. Bull Pure & Appl Sciences. 2006;25E(1):215–217.

43. Guhr T, Müller-Groeling A, Weidenmüller HA. Random-matrix theories in quantum physics: common concepts. Physics Reports. 1998;299(4):189–425.

44. Moran PA. Notes on continuous stochastic phenomena. Biometrika. 1950;37(1-2):17–23.
